# Sacrificial layer concept interface engineering for robust, lossless monolithic integration of perovskite/Si tandem solar cells yielding high fill factor of 0.813

**DOI:** 10.1186/s40580-025-00492-3

**Published:** 2025-05-27

**Authors:** Yoon Hee Jang, Youngseok Lee, Hyeon Sik Seo, Haram Lee, Kyoung-jin Lim, Jung-Kun Lee, Jaeyeong Heo, Inho Kim, Doh-Kwon Lee

**Affiliations:** 1 Advanced Photovoltaics Research Center, Korea Institute of Science and Technology (KIST), Seoul, 02792 Republic of Korea; 2 Center for Semiconductor Technology, Korea Institute of Science and Technology (KIST), Seoul, 02792 Republic of Korea; 3 PVCVD Team, R&D Center, Jusung Engineering Co., Ltd, Yongin, 17094 Republic of Korea; 4 Department of Mechanical Engineering and Materials Science, University of Pittsburgh, PA, 15260 USA; 5 Department of Materials Science and Engineering, Optoelectronics Convergence Research Center, Chonnam National University, Gwangju, 61186 Republic of Korea; 6 Division of Nano and Information Technology, KIST School, University of Science and Technology, Seoul, 02792 Republic of Korea; 7 Current Address: Cell R&D Team, Hanhwa Q CELLS, Jincheon-gun, Republic of Korea

**Keywords:** Perovskite/Si tandem solar cell, Monolithic integration, Recombination junction, Interface engineering, Sacrificial layer, PEDOT:PSS, Band alignment

## Abstract

Efficient monolithic perovskite/Si tandem solar cells require a robust recombination junction (RJ) with excellent electrical and optical properties. This study introduces an interface engineering method using an organic sacrificial layer to enable effective monolithic integration. An ultrathin layer of poly(3,4-ethylene-dioxythiophene):polystyrene sulfonate (PEDOT:PSS) is inserted between the transparent conductive oxide recombination layer and the hole transport layer (HTL) of a methylammonium lead iodide (MAPbI_3_)-based perovskite top cell. This layer restores junction functionality and enables charge transfer between sub-cells via efficient carrier recombination at the RJ, which electrically connects the two cells. Acting as a sacrificial layer, PEDOT:PSS temporarily prevents resistive SiO_*x*_ formation and improves interface quality. High-resolution transmission electron microscopy and X-ray photoelectron spectroscopy confirm suppression of SiO_*x*_ growth during HTL annealing. Moreover, the Cu-doped NiO_*x*_ HTL fabrication method proves critical, where process optimization improves electrical contact. Combined with PEDOT:PSS interface engineering, these enhancements promote efficient recombination by tuning interfacial energy levels and increasing band bending at the RJ. As a result, tandem devices comprising an aluminum back-surface field *p*-type homojunction Si bottom cell and a *p-i-n* perovskite top cell achieve 21.95% power conversion efficiency and an 81.3% fill factor —among the highest reported for monolithic perovskite/Si tandem solar cells.

**Supplementary Information:**

The online version contains supplementary material available at 10.1186/s40580-025-00492-3.

## Graphical Abstract

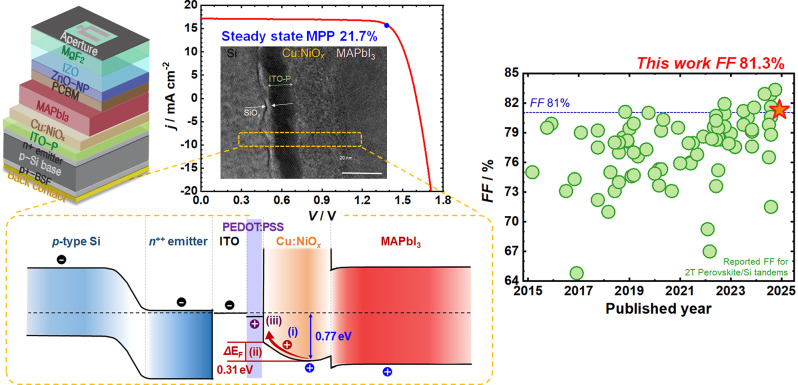

## Introduction

Crystalline silicon (c-Si) solar cells have dominated the global photovoltaic (PV) market for decades due to their high efficiency and steadily declining production costs. To further increase the contribution of PVs to global electricity generation, it is essential to lower the levelized cost of energy by improving power conversion efficiency (PCE). One of the most promising pathways to exceed the efficiency limit of single-junction (SJ) Si solar cells is through multijunction tandem architectures [1]. Among these, perovskite/Si tandem solar cells have emerged as the leading candidate, offering both high theoretical efficiency and compatibility with existing Si manufacturing. This technology has gained significant momentum and is expected to reach commercial-scale production by 2027 [2]. Over the past decade, significant progress has been made in perovskite/Si tandem solar cells [3, 4, 5, 6]. Recently, the Chinese solar technology company LONGi announced a record-breaking power-conversion efficiency of over 34% for a perovskite/Si tandem solar cell [7], which is notably higher than the current record efficiency of single-junction c-Si devices (27.3%) [8] and their theoretical efficiency limit of 29.4% [9].

Figure S1 illustrates the efficiency evolution of perovskite/Si 2-terminal (2T) tandem solar cells. In the early stages of perovskite/Si tandem research, perovskite with a *n-i-p* configuration and c-Si with a polished top surface were commonly used as the top and bottom cells, respectively, to obtain 2T tandems. A pioneering study by Mailoa et al. in 2015 [10] utilized a front-polished *n*-type homojunction Si bottom cell and a *n-i-p* configured perovskite top cell, which reached a PCE of 13.7% on a 1 cm^2^ device. In that study, a silicon-based interband tunnel junction (*n*^++^/*p*^++^ Si) was developed and investigated to facilitate charge-carrier recombination. Following this early demonstration, c-Si heterojunction (SHJ) cells—known for their high open-circuit voltage and efficiency—became the preferred bottom cell architecture in most subsequent studies. However, the intrinsic amorphous silicon (a-Si: H) layer in SHJ cells has limited thermal stability, which constrains processing temperatures to below 200 °C. As a result, considerable research has focused on developing low-temperature fabrication methods for the perovskite top cell to ensure compatibility with SHJ-based tandems [11]. However, the efficiency of *n-i-p* perovskite/SHJ tandems was limited due to parasitic absorption by the commonly used hole-transport material, 2,2’,7,7-tetrakis(N,N-di-p-methoxypheniylamine)-9,9-spirobifluorene (Spiro-OMeTAD), especially in the short wavelength (below 420 nm) and near-infrared region [12]. In order to overcome these limitations, the *p-i-n* structured semitransparent (ST) perovskite top cell emerged as a more suitable alternative for tandem applications that offers structural advantages, better manufacturing compatibility, and flexibility in functional layer selection [13, 14]. The combination of a SHJ bottom cell with a *p-i-n* ST perovskite top cell led to important breakthroughs, with tandem device efficiencies approaching 30% [15, 16, 17, 18, 19]. Continued improvements have been driven by advances in functional layer materials, deposition techniques for perovskite top cells on textured Si surfaces, and refined optical designs [3, 5, 6, 20, 21, 22, 23].

Most research on 2T perovskite/Si tandem solar cells to date has focused on enhancing overall efficiency by optimizing the individual subcells. However, in the race to achieve higher efficiencies, relatively little attention has been given to the integrated junction interface despite its crucial role in determining the electrical and optical performance of monolithic tandem architectures. Mailoa et al. [10] investigated the *n*^++^/*p*^++^ Si tunnel junction and reported that inserting a thin intrinsic a-Si:H layer between the heavily doped layers could suppress dopant diffusion during post-deposition annealing and preserve junction conductivity. Inspired by this work, several follow-up studies have explored the development of nanocrystalline Si (nc-Si:H) tunneling layers to improve both the optical and electrical properties of the interconnecting junction [24, 25, 26].

In contrast, the role of transparent conductive oxide (TCO)-containing interlayers has received little attention, even though such layers have already shown promise in monolithic tandem solar cells. Only a few studies have investigated suitable materials and deposition conditions for TCO-based recombination layers to address associated optical and electrical losses [27, 28, 29]. For example, Hoye et al. [30] demonstrated that an indium tin oxide (ITO) recombination layer can protect the Si surface from oxidation during the deposition of nickel oxide (NiO_*x*_) hole transport layers (HTLs). In contrast to previous studies, we found that a SiO_*x*_ charge-extraction barrier readily forms during the deposition of the HTL when high-temperature processing is involved, which can lead to deterioration of the ITO-based recombination junction (RJ). To enable robust monolithic tandem integration featuring ITO and copper-doped NiO_*x*_ (Cu: NiO_*x*_) as the recombination contact, we developed a facile and universal method based on sacrificial interface engineering. Specifically, we demonstrated that the deliberate introduction of an ultrathin organic layer, poly(3,4-ethylenedioxythiophene):polystyrene sulfonate (PEDOT:PSS), effectively suppresses SiO_*x*_ formation on the Si surface during the formation of the Cu:NiO_*x*_ HTL.

Moreover, this ultrathin PEDOT:PSS layer enables a tunnel junction that facilitates charge recombination, driven by the strong built-in electric field arising from Fermi-level differences. As a proof-of-concept, we fabricated monolithic tandem solar cells using high-temperature-compatible *p*-type homojunction c-Si bottom cells with an aluminum back-surface field (Al-BSF) paired with a *p-i-n* structured perovskite (CH_3_NH_3_PbI_3_; MAPbI_3_; MAPI) top cell. While current commercial technologies primarily use passivated emitter and rear cell (PERC) or tunnel oxide passivated contact (TOPCon) structures [2], the Al-BSF configuration was chosen here for its simplicity and structural similarity to PERC, which differs mainly in rear-side passivation. Therefore, the Al-BSF cell provides an ideal model platform to explore tandem integration.

By combining this architecture with our sacrificial interface engineering, we achieved tandem cell efficiencies of up to 21.95% and a steady-state efficiency of 21.7%. The robust junction interface enabled by the PEDOT:PSS sacrifice layer also led to high device reproducibility and a high fill factor (*FF*). Notably, one of the tandem devices exhibited a *FF* of 81.3%, which is among the highest reported for 2T perovskite/Si tandem solar cells employing an Al-BSF bottom cell. Based on optical simulations, we estimate that a PCE of 25.4% is achievable with a state-of-the-art Al-BSF c-Si bottom cell, with even higher potential efficiencies expected when integrating current mainstream PERC or TOPCon technologies. Further improvements are anticipated through structural optimization of the perovskite top cell, including composition tuning, incorporation of advanced charge transport layers, and implementation of refined interface-engineering techniques [31, 32, 33].

## Methods

### Fabrication of Al-BSF *p*-type homojunction silicon solar cells

For the fabrication of Al-BSF Si subcells, (100)-oriented *p*-type CZ-Si wafers with a resistivity of 1 − 5 Ω·cm and a thickness of 525 μm were cleaned using standard RCA1 and RCA2 solutions. A 2-μm-thick Al layer was deposited on the rear side of the Si wafers using e-beam evaporation, followed by spin-coating of a phosphorus spin-on-dopant (SOD P507, Filmtronics) onto the polished front surface. The co-diffusion process, which simultaneously formed the *n*^++^ emitter and *p*^+^ Al-BSF, was carried out using a rapid thermal annealing system at 900 °C for 42 s. After annealing, phosphor-silicate glass and excess Al on both sides of the Si wafer were removed with a hydrofluoric acid solution, followed by RCA cleaning. ITO thin films for the recombination contacts were then deposited on the *n*^++^ emitter via radio frequency (RF) sputtering at room temperature (50 W) under a mixed Ar/O_2_ atmosphere. The deposition was conducted at a working pressure of 1 mTorr, with the oxygen content varying between 0 and 5% to optimize film properties (Fig. S2). The ITO films deposited without oxygen appeared brownish and exhibited low optical transparency. The highest Haacke figure of merit was achieved at 0.75% oxygen content, which was thus used for all subsequent ITO depositions. The thickness of the ITO layer was maintained at 20 nm. Subsequently, the ITO films were annealed at 400 °C for 20 min in forming gas using a tube furnace to enhance crystallinity. A square photoresist mask (0.36 cm^2^) was then patterned on the ITO layer using photolithography. The unmasked regions of the ITO and underlying Si emitter were selectively etched using hydrochloric acid and sulfur hexafluoride reactive ion etching for cell edge isolation. The Si subcells were finalized by depositing an additional 2-µm-thick Al rear electrode via e-beam evaporation. These subcells were subsequently used as bottom cells for tandem device fabrication. For performance characterization of the Si subcells, a 2-µm-thick Ag front grid electrode was thermally evaporated onto the ITO-coated Si emitter.

### Fabrication of semitransparent perovskite solar cells

ST perovskite SJ devices were fabricated on ITO-coated glass substrate (*R*_□_ ~9 Ω, AMG Tech.). The substrates were sequentially cleaned via ultrasonication in acetone, ethanol, and isopropyl alcohol, followed by an UV-ozone surface treatment for 20 min to induce surface hydrophilicity. A 5 mol% Cu:NiO_*x*_ precursor solution was prepared using nickel (II) nitrate hexahydrate (Aldrich), copper (II) nitrate trihydrate (Sigma-Aldrich), and ethylenediamine (Sigma-Aldrich) in anhydrous ethylene glycol (Sigma-Aldrich) at a total concentration of 1 M. This solution was spin-coated onto the ITO substrate at 2000 rpm for 90 s, then annealed sequentially at 100 °C for 5 min and 300 °C for 1 h on a hot plate. After annealing, the Cu:NiO_*x*_ films were rapidly cooled and immediately transferred to an N_2_-filled glove box for further processing. The perovskite layer was formed from a 1.4 M non-stoichiometric solution of methylammonium iodide (MAI, Dyesol) and lead (II) iodide (PbI_2_, Alfa Aesar) in a molar ratio of MAI/PbI_2_ = 1.04. The solvent system consisted of *γ*-butyrolactone (Aldrich) and dimethyl sulfoxide (Sigma-Aldrich) (7:3 v/v). The solution was filtered through a 0.45 μm PTFE syringe filter and deposited onto the Cu:NiO_*x*_ layer via a two-step spin-coating process: 1000 rpm for 10 s and 4500 rpm for 20 s. During the last 5 s of the second step, chlorobenzene was dropped as an antisolvent to induce rapid crystallization.

The resulting film was annealed at 100 °C for 10 min to form crystalline MAPI. A 20 mg ml^–1^ solution of [6, 6]-phenyl-C_61_-butyric acid methyl ester (PCBM, 1-Material Inc.) in chlorobenzene was then spin-coated on the perovskite film using the same spin-coating process with perovskite. To prevent plasma damage from the subsequent top-electrode deposition, a ZnO-nanoparticle (ZnO-NPs) layer (Nanograde N-10) was deposited atop the PCBM layer [34]. A 100-nm-thick indium zinc oxide (IZO) film was then deposited as the top TCO electrode using RF magnetron sputtering at 50 W and an oxygen flow rate of 0.1 sccm. A shadow mask was used during the deposition to define the cell area (5.5 mm × 5.5 mm). Finally, Ag grids were thermally evaporated as the front contact, and the active area of each ST perovskite device was defined as 0.2637 cm^2^. To avoid overestimation of the photocurrent, an aperture mask with identical dimensions to the shadow mask was used during the measurements.

### Fabrication of monolithic Perovskite/Si tandem devices

An Al-BSF *p*-type homojunction Si solar cell and a planar *p-i-n* perovskite solar cell (PSC) were electrically connected through a 20-nm-thick ITO RJ layer. The Si bottom cell was laser-cut into 30 × 30 mm^2^ pieces, and the perovskite top cell was subsequently fabricated directly onto the ITO RJ using the same procedure as for the ST perovskite SJ device. To complete the tandem device stack, an IZO/Ag top contact was deposited using the same method as described previously, followed by the thermal evaporation of a 105-nm-thick MgF_2_ antireflection (AR) coating. The active area of the tandem device was defined by the area of the top TCO deposited through the shadow mask. To suppress undesired dark-current generation, the perimeter of the active area was masked during the measurement.

### Device characterization

The current density–voltage (*j*–*V*) measurements were performed using a Keithley model 2400 source measure unit and a class-A solar simulator (Yamashita Denso, YSS-50 A) equipped with a 180 W xenon lamp. For the semitransparent (ST) perovskite measurement, the illumination intensity was calibrated to the AM 1.5G one-sun condition (100 mA cm^− 2^) using a National Renewable Energy Laboratory (NREL)-calibrated, KG5-filtered Si reference cell (PV Measurement, Inc.). For tandem device measurements, due to the spectral mismatch between the simulator and the standard AM 1.5G spectrum, the light intensity was calibrated using Si reference cells with different window materials (e.g., KG5 or BK7), depending on which subcell limited the current generation. Both ST perovskite and tandem solar cells were measured under reverse and forward scan modes with a 100 ms delay between data points. External quantum efficiency (EQE) measurements were carried out using an incident photon-to-current conversion measurement system (G1218a, PV Measurement, Inc.) and QUANTX-300 quantum-efficiency measurement solution (Newport). The EQE of the Si bottom cell in tandem devices was extracted by saturating the perovskite top cell using a 520 nm collimated laser diode (CPS 520, Thorlabs, Inc.), while the EQE of the perovskite top cell was measured separately on a tandem-like stack fabricated on a *n*-type Si substrate. The surface morphology of the perovskite film was examined using field-emission scanning electron microscopy (FE-SEM, FEI Inspect F, or Hitachi Regulus 8230). High-resolution transmission electron microscopy (HR-TEM) and high-angle annular dark-field scanning transmission electron microscopy (HAADF-STEM) were performed using an FEI Titan 80–300 microscope. Elemental mapping via energy-dispersive X-ray spectroscopy (EDS) was conducted using a FEI Talos F200X. The tunnel junction characteristics were analyzed using ultraviolet photoelectron spectroscopy (UPS, ESCALAB250Xi-X-ray Monochromator, Theromo-Scientific) and X-ray photoelectron spectroscopy (XPS, PHI 5000 VersaProbe, ULVAC PHI). The optical properties were measured via ultraviolet-visible-near-IR spectroscopy (UV-Vis-NIR, Agilent Cary 5000).

### Optical simulations

The optical absorptance of each layer in the perovskite/planar Al-BSF Si tandem solar cells is simulated by using a commercial software package based on the combined optical models of ray optics and wave optics (CROWM, Ljubljana University). All layers in the perovskite/Al-BSF Si tandem cells were considered coherent except the Si wafer. In order to take the light scattering at the rear side of a Si bottom cell into consideration, the morphology of the Al-BSF (analyzed via atomic force microscopy) was imported into the simulation software (Fig. S21). The refractive indices (*n*) of the constituent materials were determined for the optical calculations using spectroscopic ellipsometry in the wavelength range of 350 to 1200 nm (Fig. S22). The equivalent current densities (*j*_ph_) of the subcells were calculated from the absorptance spectra of each layer, and the total reflection spectra of the tandem cells were also simulated. In order to account for the electrical losses of the Si bottom cell, the internal quantum efficiency (IQE) was determined from the measured EQE and the reflectance of the SJ Al-BSF Si solar cells with an ITO top electrode.

Furthermore, to provide a design guide to improve tandem cell performance, additional simulations were performed using the CROWM software package while varying the thicknesses of functional layers such as ZnO-NP, PCBM, Cu:NiO_*x*_, and the front and interlayer TCOs (IZO and ITO). Finally, the matching currents in the tandem cells were examined by varying the thickness of perovskite and the back surface recombination velocity (BSRV) values of the Si subcells. By locating the maximum matching currents, we were able to provide predictions of the practical efficiency limits for perovskite/Al-BSF Si tandem solar cells.

## Results and discussion

### Semitransparent single-junction perovskite solar cells

**Fig. 1:**
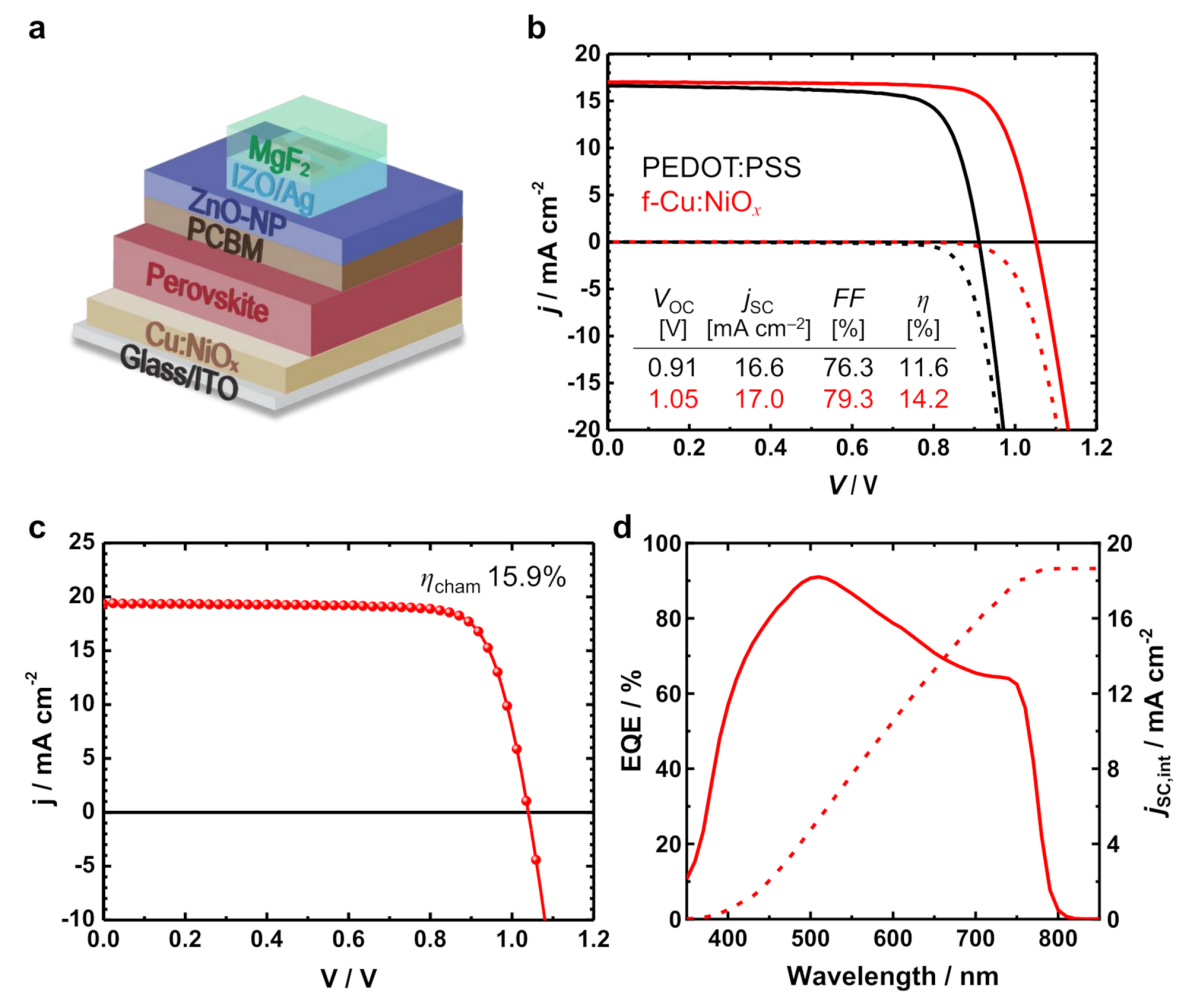
(**a**) Illustration of a semitransparent perovskite solar cell (ST-PSC) device with the structure glass/ITO/Cu:NiO_*x*_/MAPI/PCBM/ZnO-NP/IZO/Ag/MgF_2_. (**b**) *j*–*V* characteristics of representative ST-PSCs with different HTLs, PEDOT:PSS and f-Cu:NiO_*x*_ (without an antireflection (AR) layer). (**c**) *j*–*V* curve and (d) EQE spectrum of the best f-Cu:NiO_*x*_-based ST-PSC with an MgF_2_ AR layer. Note that all *j*–*V* and EQE curves were measured in substrate configuration

In order to successfully implement a top cell architecture that can be directly applied to monolithic tandem solar cells, we first fabricated SJ ST-PSCs on ITO-coated glass substrates in a *p-i-n* configuration. Figure 1a displays a schematic of the ST device with a structure of ITO/Cu:NiO_*x*_/MAPI/PCMB/ZnO-NP/IZO/Ag-grid/MgF_2_, where the ZnO-NP layer was deposited to protect the underlying perovskite/PCBM layers against plasma damage during the IZO sputtering process [34]. The MAPI films were deposited using anti-solvent-assisted crystallization processes on a Cu:NiO_*x*_ HTL. NiO_*x*_ has been widely used in PSCs as the HTL due to its wide bandgap and *p*-type properties and its high tolerance to thermal stress [35, 36, 37]. However, the NiO_x_-based devices typically show a low level of open-circuit voltage (*V*_OC_) compared to those with organic HTLs, which is reported to be due to defect-associated interfacial recombination [38, 39].

In order to improve *V*_OC_ and the device performance of the PSCs based on the pristine NiO_*x*_ HTL, slight Cu-doping was used [40, 41]. Note that the Cu:NiO_*x*_ layers were prepared in two different ways. Initially, the film was annealed at 300 °C and slowly cooled after spin-coating the sol-gel precursor solution. In the optimized process, the film was pre-annealed first at 100 °C and annealed again at 300 °C. This was followed by rapid quenching. As shown in Fig. S3, this optimization resulted in a fine-grained Cu:NiO_*x*_ morphology. Accordingly, the Cu:NiO_*x*_ prepared using the initial method is referred to as c-Cu:NiO_*x*_ (coarse Cu-doped NiO_*x*_), while that prepared using the optimized method is referred to as f-Cu:NiO_*x*_ (fine Cu-doped NiO_*x*_). The impact of the preparation method on device performance is discussed in detail below.

As a preparatory experiment, we also investigated the effects of Cu-doping on the devices performance of opaque PSCs (Fig. S4a). As shown in Fig. S4b, compared to pristine NiO_*x*_ film, the Cu:NiO_*x*_ film showed a relatively dense and uniform morphology with a smaller particulate size. The impact of this on the growth of perovskite films is shown in Fig. S4c. The grain sizes of perovskite films deposited on NiO_*x*_ and Cu:NiO_*x*_ HTLs were (240 ± 60) nm and (330 ± 70) nm, respectively, which confirms the facilitated grain growth of perovskite films on Cu:NiO_*x*_ HTL. The resulting *j*–*V* characteristics of ST-PSCs using pristine NiO_*x*_ and Cu:NiO_*x*_ are shown in Fig. S4d. A significant performance improvement, not only in terms of *V*_OC_ but even more pronounced in short-circuit current density (*j*_SC_) and *FF*, was observed when Cu:NiO_*x*_ was used as an HTL. The overall improved device characteristics can be interpreted as a consequence of the improved bulk conductivity of Cu:NiO_*x*_ [40] and/or the interface electrical properties between Cu:NiO_*x*_ and the perovskite layer, which, in turn, can be attributed to the dense Cu:NiO_*x*_ film and favorable growth of perovskite on dense HTL.

It has been reported that the inorganic NiO_*x*_-based HTL offers advantages over the conventional PEDOT:PSS HTL in tandem applications, including enhanced stability and *V*_OC_ [30, 37]. Figure 1b shows the *j*–*V* curves of representative ST-PSC devices based on PEDOT:PSS and Cu:NiO_*x*_ HTLs for comparison. As shown in Fig. 1b and Fig. S5, the efficiency of the ST-PSCs with inorganic Cu:NiO_*x*_ HTL was greatly improved compared to that of the PEDOT:PSS-based devices. This effect is mainly attributed to the significant gain in *V*_OC_ (*ΔV*_OC_ ≈ 120 mV on average). The larger *V*_OC_ of Cu:NiO_*x*_-based devices could be attributed to two different contributions: (1) reduced potential loss at the HTL and perovskite interface thanks to energetically favorable energy-level alignment [40, 41]; (2) suppressed grain-boundary recombination thanks to the formation of larger grains of MAPI on Cu:NiO_*x*_ than those on PEDOT:PSS (Fig. S6) [42, 43, 44]. The inorganic Cu:NiO_*x*_ HTL also noticeably improved the device stability. As shown in Fig. S7, Cu:NiO_*x*_-based ST-PSC devices exhibit much better device storage stability under an inert atmosphere in a glove box (retaining over 95% of the initial efficiency after 1320 h) compared to PEDOT:PSS devices (which retained less than 80%). This improvement is believed to be due to the replacement of acidic and hygroscopic PEDOT:PSS with Cu:NiO_*x*_, thus preventing corrosion of the ITO electrode [45]. Figure 1c shows the best performance achieved by the Cu:NiO_*x*_ HTL-based ST-PSC measured in the substrate configuration. Specifically, an efficiency of 15.9% with a *V*_OC_ of 1.04 V, *j*_SC_ of 19.4 mA cm^–2^, and *FF* of 79.0% was achieved, representing a competitive performance among NiO_*x*_-based ST-PSCs in the substrate configuration (Table S1). Figure 1d shows the EQE spectrum of the ST-PSC, where the integrated current density (*j*_SC, int_) calculated from the EQE was 18.7 mA cm^–2^, slightly lower than the value obtained from the *j*–*V* measurement. The corresponding PCE calculated using *j*_SC, int_ was 15.4%.

### Monolithic integration via modified recombination contact

**Fig. 2:**
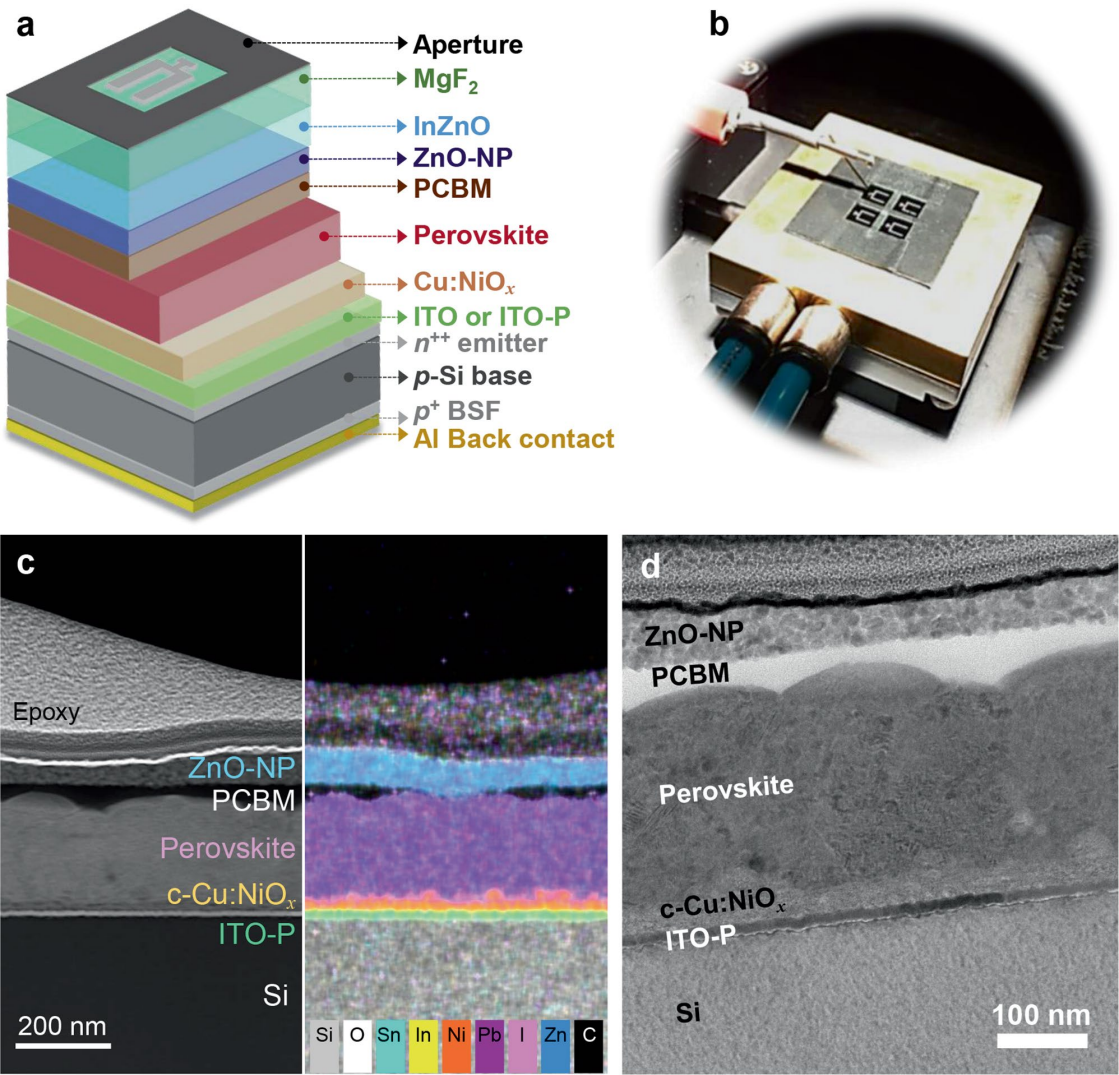
(**a**) Schematic of the perovskite/Al-BSF *p*-type homojunction Si monolithic tandem solar cell (not to scale). (**b**) Photograph of real tandem devices being measured under simulated AM 1.5G 1-sun-equivalent illumination. (**c**) HAADF-STEM, together with elemental mapping images, and (**d**) HR-TEM image of the tandem device

To fabricate the monolithic perovskite/Si tandem solar cells, we modified a standard Al-BSF c-Si solar cell for use as a bottom cell. The front SiN_*x*_ layer on the planar Si phosphorus-diffused *n*^++^ emitter (which serves as an antireflection and passivation layer) was replaced with an ITO recombination layer ca. 20 nm in thickness. Unlike a previous report [30], the optimal content of oxygen (a mixing ratio of 0.75%) was introduced during the sputter deposition of ITO to obtain highly transparent and conductive films (Fig. S2). Subsequently, the ITO layer and the Si *n*^++^ emitter—except the cell area—were removed through a successive etching process to avoid shunting problems at the cell edges and to accurately define the cell area. A semitransparent perovskite top cell was then prepared on top of the Si bottom cell, while both subcells shared the ITO recombination layer. The completed monolithic tandem device has a structure of Al/Al-BSF/*p*-Si/*n*^++^-Si/ITO/Cu:NiO_*x*_/MAPI/PCMB/ZnO-NP/IZO/Ag-grid/MgF_2_, as schematically illustrated in Fig. 2a. The layered structure of the tandem device was confirmed using HAADF-STEM along with EDS elemental mapping and HR-TEM– see Fig. 2c–d. The photograph displayed in Fig. 2b shows real perovskite/Si tandem devices, where an aperture mask with the same dimension as the top TCO layer was used to define the active area (0.2637 cm^2^).

In the monolithic integration of two subcells connected in series, an electrically and optically lossless recombination junction is essential to obtain high-efficiency tandem solar cells. In order to assess the quality of the recombination junction, initial experiments examined the effect of the ITO recombination layer thickness on tandem device performance. However, the monolithic perovskite/Si tandem solar cells with ITO/Cu:NiO_*x*_ RJ consistently showed high series resistance (*R*_s_)—regardless of ITO thickness (Fig. S8). These results suggest that the resistive behavior originates not from the intrinsic properties of the sputtered ITO layer but rather from the mismatched interface between ITO and Cu:NiO_*x*_ layers.

To reduce the interface resistance of the tandem devices, we considered modifying the interface between the ITO and Cu:NiO_*x*_ layers using PEDOT:PSS. Although PEDOT:PSS is known for its acidic and hygroscopic nature (which can be detrimental to device stability), we chose it as an interface modifier based on our prior success in monolithic integration using TCO/PEDOT:PSS junctions in perovskite/CuInSe₂ [34] and perovskite/Si tandem solar cells (Fig. S9). PEDOT:PSS features high optical transparency and decent conductivity, which are desirable properties for an interlayer in monolithic tandem devices [46, 47]. Nevertheless, to minimize potential drawbacks, such as optical transmittance loss due to the insertion of an additional layer and stability issues from PEDOT:PSS acidity, we deliberately changed the PEDOT:PSS solution with methanol to reduce the layer thickness to just a few nanometers (ca. 2.7 nm, as measured by ellipsometry). The thin layer was eventually removed (burned) during the post-annealing process of the Cu:NiO_*x*_ HTL. As a result, we define PEDOT:PSS here as a sacrificial layer that does not cause optical losses (Fig. S10), which will be discussed in more detail in the following sections.

**Fig. 3:**
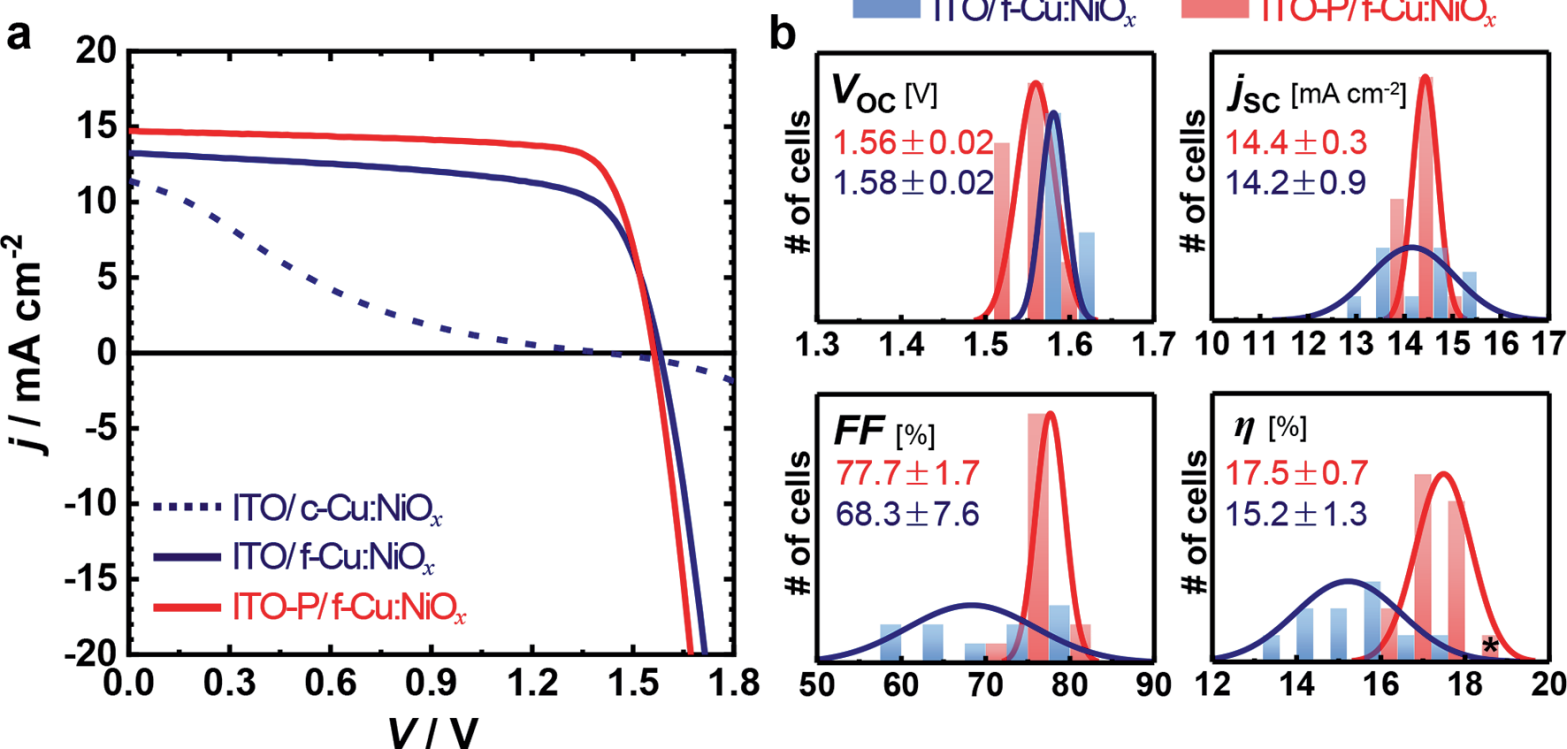
PV performance of the perovskite/Al-BSF Si monolithic tandem devices without MgF_2_ AR layer and different recombination junctions (RJs), ITO-P/f-Cu:NiO_*x*_ (PEDOT:PSS-engineered) and ITO/f-Cu:NiO_*x*_ (control): (**a**) Typical *j*–*V* curves, (**b**) statistic representation of PV parameters of 16 and 9 tandem devices employing PEDOT:PSS-engineered (in red) and control (in blue) RJs, respectively. The asterisk in part b represents the best-performing device without an AR layer

Figure 3a shows the *j*–*V* curves of Si/perovskite tandem devices featuring three types of recombination junctions: the control RJ (ITO/f-Cu:NiO_*x*_), the PEDOT:PSS-engineered RJ (ITO-P/f-Cu:NiO_*x*_), and an RJ using initially processed Cu:NiO_*x*_ (c-Cu:NiO_*x*_). Notably, further optimization of the Cu:NiO_*x*_ deposition process effectively mitigated detrimental junction damage, even in the absence of PEDOT:PSS-engineering. This optimized procedure significantly improved the morphology of the Cu:NiO_*x*_ film (Fig. S3), which enhanced not only the intrinsic electrical properties of Cu:NiO_*x*_ but also its interfacial contact with adjacent layers. The beneficial impact of this optimization is further evidenced by the improved PV performance of ST-PSCs, where f-Cu:NiO_*x*_ exhibited improved PV parameters compared to c-Cu:NiO_*x*_ (Fig. S11). Interestingly, while PEDOT: PSS-engineering showed a minimal effect in the case of f-Cu:NiO_*x*_, it substantially reduced *R*_s_ when combined with c-Cu:NiO_*x*_. This finding indicates that the PEDOT:PSS layer facilitates intimate contact at the ITO/HTL interface. Compared to the control RJ, the PEDOT:PSS-engineered RJ exhibited a lower *R*_s_ (resulting in enhanced *FF* and PCE), as shown in Fig. 3a. Moreover, the introduction of PEDOT:PSS enabled robust processability, leading to narrow PV parameters distributions and improved device reproducibility (Fig. 3b). This effect is especially pronounced in tandem cells that use c-Cu:NiO_*x*_ as HTL (Fig. S12). Collectively, these results clearly demonstrate that ultrathin PEDOT:PSS-engineering is highly effective in mitigating interfacial resistance between the top and bottom cells, which enables a robust and lossless recombination junction in monolithic perovskite/Si tandem devices.

**Fig. 4:**
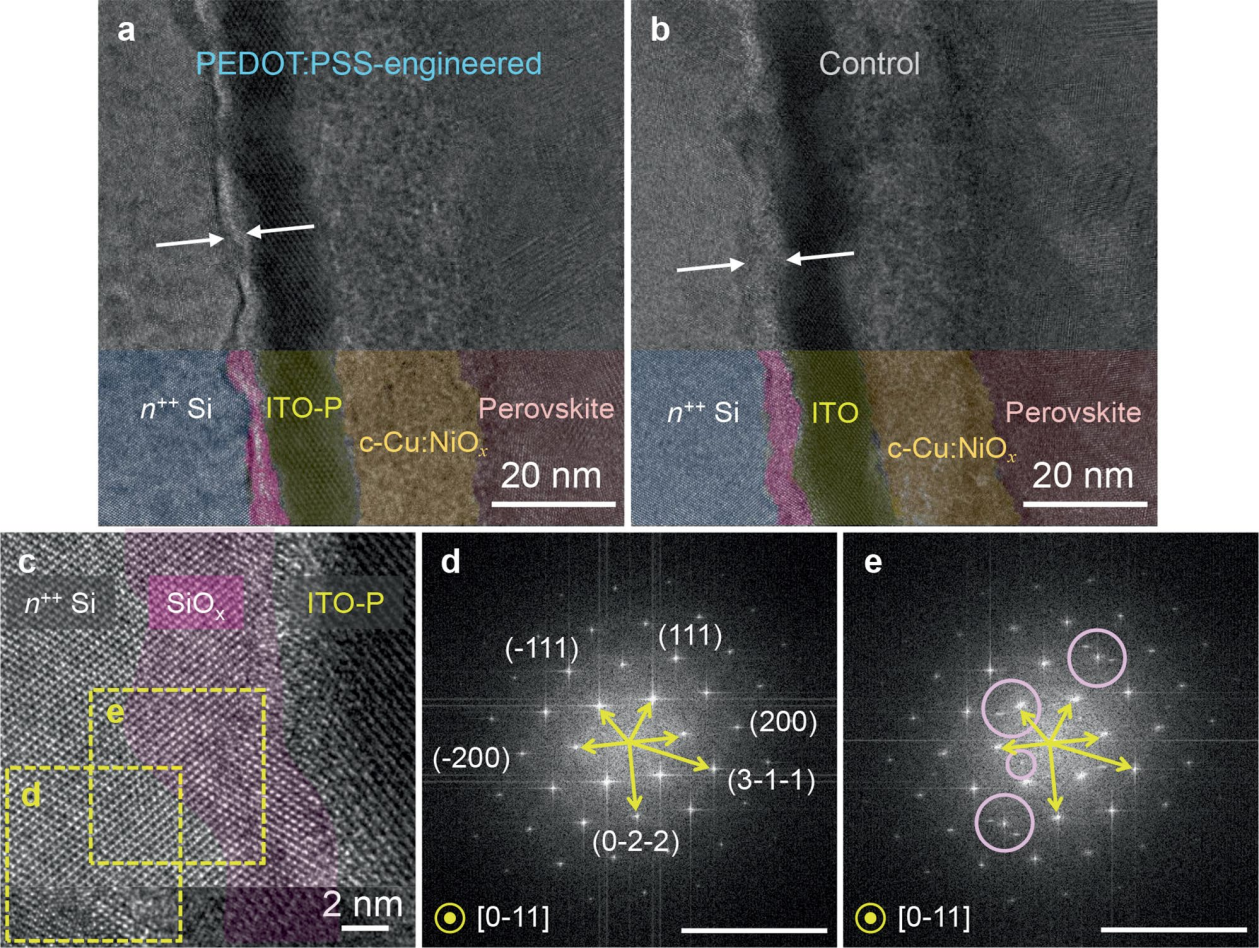
HR-TEM images of a perovskite/Al-BSF Si tandem device near the recombination junctions: (**a**) Si/ITO-P/c-Cu:NiO_*x*_/perovskite and (**b**) Si/ITO/c-Cu:NiO_*x*_/perovskite. The white arrows in parts a and b indicate amorphous SiO_*x*_ layers of different thicknesses between *n*^++^ Si and ITO, as evidenced by XPS and STEM-EDS mapping in Fig. S13 and S14, respectively. (**c**) Enlarged view near the Si/ITO-P interface region and (**d**) and (**e**) the corresponding fast Fourier-transform images for the marked areas in part c. Note that the circles in part e show the appearance of additional diffraction patterns other than Si in the SiO_*x*_ layer

To microscopically and crystallographically investigate the origin of resistive integration of the subcells before interface engineering with the sacrificial PEDOT:PSS layer and better understand how this interface engineering mitigates the detrimental interface resistance at RJ, we carried out cross-sectional HR-TEM, STEM-EDS elemental mapping, and XPS depth profile studies. Figure 4a–c depicts the layered structures near the control and PEDOT:PSS-engineered RJs. A distinct amorphous layer at the interface between the Si *n*^++^ emitter and ITO is clearly observed—as indicated by arrows. The reciprocal image corresponding to the emitter region (Fig. 4d), obtained by fast Fourier transform (FFT), shows a series of crystallographic planes perpendicular to the zone axis [0–11] of cubic Si (Fd-3 m). In addition, the FFT image taken across the Si emitter and the amorphous regions (Fig. 4e) reveals additional diffraction patterns that do not belong to cubic Si. To identify this amorphous layer, we analyzed both RJs using XPS depth profiling (Ar^+^ ion etching with 2 kV). Fig. S13 shows the XPS elemental depth profiles and high-resolution Si 2p core-level emission spectra as a function of sputter etching time for both the control and PEDOT:PSS-engineered RJs. As in the Si 2p spectra in Fig. S13b–c, in addition to the bulk Si peak (Si 2p_1/2_ and Si 2p_3/2_) at ca. 99.4 eV, an emission peak associated with oxidized silicon species (i.e., SiO_*x*_) appear at a binding energy of ~ 102.5 eV [48], regardless of whether PEDOT:PSS-engineering is applied. Based on this observation, the amorphous layer seen in the HR-TEM images is identified as SiO_*x*_. The amorphous SiO_*x*_ layer was confirmed via EDS elemental mapping of HAADF-STEM images (Fig. S14), where oxygen was found to coexist with silicon in the region beneath the ITO RJ and on the Si layer.

It is worth noting in Fig. 4a–b that the thickness of the SiO_*x*_ layer in the control RJ, 4.5 ± 0.4 nm, was ca. 1.7 times thicker than that of the PEDOT:PSS-engineered RJ, 2.7 ± 0.1 nm. This observation is in good agreement with the XPS depth profile analysis. As marked in Fig. S13a, the SiO_*x*_ signals from the control RJ were detected over an etching period that was almost double that of the PEDOT-treated junction. The SiO_*x*_ layer formed in the control RJ was thus estimated to be 1.5 − 2 times thicker than that of PEDOT: PSS-engineered RJ. From these XPS and HR-TEM results, we can deduce that the introduction of an ultrathin PEDOT:PSS layer can impede the formation of an insulating SiO_*x*_ layer at the interface between the Si bottom cell and the ITO recombination layer during the top cell processes, which alleviates the resistive nature of the RJ. Interestingly, the ultrathin PEDOT:PSS layer was not clearly observed—even at the sufficiently high resolution of the TEM studies, while only a trace amount of sulfur, which is a component of PEDOT:PSS, was detected by the EDS elemental line scan, as shown in Fig. S15. Therefore, it can be inferred that most, if not all, of the PEDOT:PSS layer decomposed during the sol-gel process of Cu:NiO_*x*_ under annealing at 300 °C in air. In other words, the ultrathin PEDOT:PSS layer acts as a sacrificial layer. To examine the effect of the PEDOT:PSS layer, which was shown to effectively inhibit the formation of the resistive SiO_*x*_ layer, on solar cell devices, we fabricated the SJ PSC test devices with and without PEDOT: PSS-engineering. To simplify the interpretation, we used a phosphor-diffused *n*-type Si wafer with a Ti/Ag back contact and a 20-nm-thick ITO front contact (Fig. S16a). Given the relatively low contact resistance of the Ti/Ag back contact and the IZO/Ag front contact, the RJs of *n*^*++*^ Si/ITO/HTLs are supposed to determine the *R*_s_ and, hence, *FF* of the test devices. The device with PEDOT: PSS-engineered interface exhibits a considerably lower *R*_s_, which results in a significantly improved *FF* (72.2%) compared to the control device (Fig. S16b). These results, combined with the TEM and XPS analyses, unambiguously demonstrate that interface engineering using the PEDOT:PSS sacrificial layer is critical to resolving the interfacial resistance problem of the Si/ITO/Cu:NiO_*x*_ junction.

**Fig. 5:**
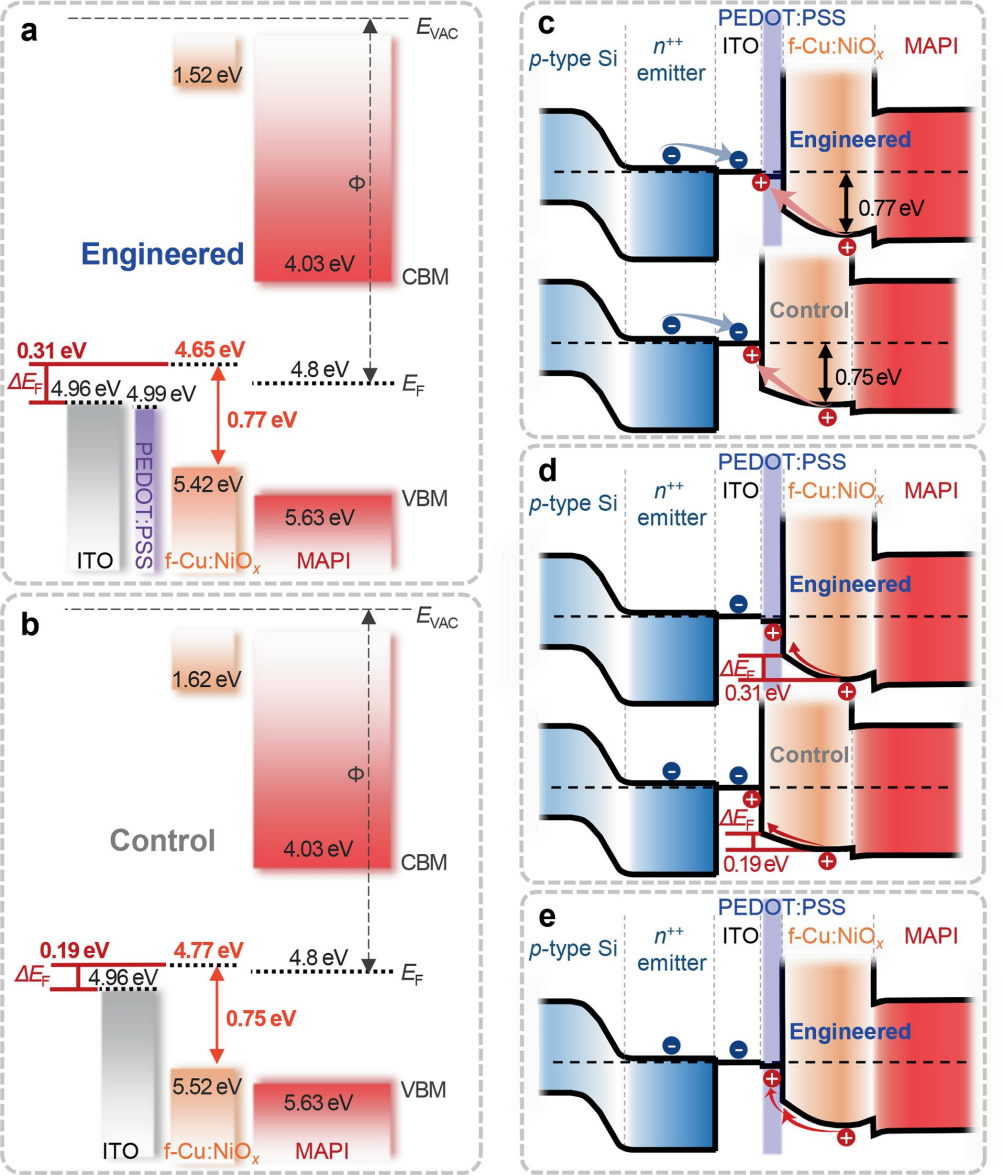
Electronic energy band diagram of perovskite/Al-BSF Si tandem devices with different recombination junctions (RJs), (**a**) ITO-P/f-Cu: NiO_*x*_ (engineered) and (**b**) ITO/f-Cu:NiO_*x*_ (control), illustrating various aspects associated with charge recombination at RJs under short-circuit condition: (**c**) thermodynamic, (**d**) kinetic, and (**e**) band-alignment perspectives

The charge recombination properties at the integration junction are among the most critical factors for achieving high efficiency in tandem devices. To understand how interface engineering via the PEDOT:PSS sacrificial layer impacts charge recombination, we examined the interfacial band alignment at the tandem junction. UPS measurements were conducted on layer stacks that were prepared in the same sequence as in the actual tandem device (i.e., Si/ITO, Si/ITO-P, Si/ITO/Cu:NiO_*x*_, and Si/ITO-P/Cu:NiO_*x*_). From these measurements, the work function and valence band (VB) onset values were extracted from the secondary electron cutoff and VB-edge regions, respectively (Fig. S17). Based on the UPS data, energy band diagrams under short-circuit conditions were constructed for tandem devices with both the control and PEDOT:PSS-engineered RJs (Fig. 5a–b and Fig. S18). The band alignment analysis suggests three mechanisms that may explain the observed charge recombination. First, from a thermodynamic perspective (Fig. 5c), the recombination rate is expected to increase as the energy level offset at the junction interface decreases. However, our system showed negligible differences in energy-level alignment between ITO/Cu:NiO_*x*_ (0.75 eV) and ITO-P/Cu:NiO_*x*_ (0.77 eV), which indicates that the PEDOT:PSS layer did not significantly impact the thermodynamic recombination rate. We then considered the impact of band bending on charge recombination from a kinetic perspective (Fig. 5d). Differences in the Fermi level (*E*_F_) or work function (*Φ*) between adjacent layers result in band bending under short-circuit conditions, which in turn induces electric fields in the space charge region. These electric fields provide a driving force for charge recombination. In our case, the band bending at the ITO-P/Cu:NiO_*x*_ interface (*ΔE*_F_ of 0.31) was significantly stronger than that at the ITO/Cu:NiO_*x*_ interface (*ΔE*_F_ of 0.19). This finding suggests enhanced electric fields and, consequently, more efficient charge recombination.

Furthermore, interface engineering with PEDOT:PSS resulted in a favorable stepwise band structure, which facilitates charge extraction at the RJ (Fig. 5e). In summary, the ultrathin PEDOT:PSS layer provides two key benefits: First, it enhances band bending which promotes efficient charge recombination at the junction. Second, it improves band alignment, which facilitates effective charge extraction. Together, these effects contribute to a low-loss recombination junction within the tandem architecture.

### Current matching for monolithic Perovskite/Si tandem solar cells

Previous studies have clearly demonstrated that achieving current matching between subcells is critical to maximizing both the matched current and PCE in monolithic tandem devices. To address this, widely adopted methods include tuning the perovskite layer thickness and bandgap, as well as optimizing optical interference within the device structure [28, 49, 50, 51, 52, 53, 54]. In this paper, we focused on adjusting the light absorption in the perovskite top cell by varying the thickness of the MAPI layer while keeping the thicknesses of all other layers fixed. The MAPI thickness was finely controlled by adjusting the concentration of the perovskite precursor solutions, which resulted in layers with measured thicknesses of 220 ± 11, 250 ± 9, and 270 ± 10 nm, as confirmed by cross-sectional FE-SEM (Fig. S19a). The *j*–*V* curves of ST-PSCs with varying MAPI thicknesses (Fig. S19b and Table S2) revealed a systematic increase in *j*_SC_ with increasing thickness. Corresponding EQE and transmittance spectra (Fig. S19c) further revealed that the spectral response of the semitransparent top cells was significantly influenced by optical interference effects, which are strongly dependent on perovskite thickness. Based on these findings, we fabricated monolithic perovskite/Al-BSF Si tandem solar cells using ST-PSCs with different MAPI thicknesses (bandgap of 1.6 eV, Fig. S19d), with the goal of achieving optimized, lossless current matching between the subcells.

**Fig. 6:**
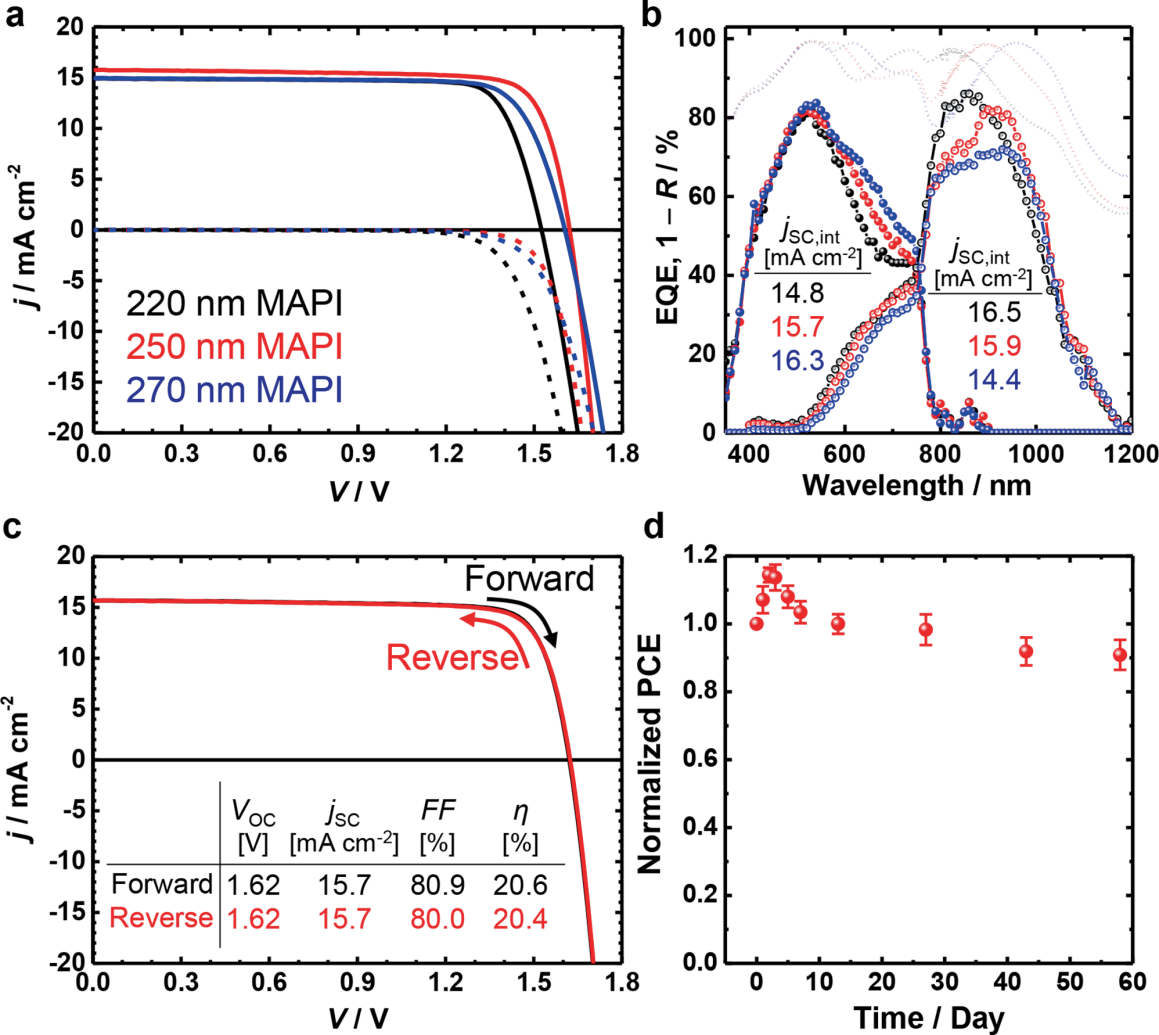
(**a**) Representative (performance closest to the average) *j*–*V* curves of interface-engineered perovskite/Al-BSF Si monolithic tandems (Si/ITO-P/f-Cu:NiO_*x*_/MAPI/PCBM/ZnO-NP/IZO/Ag/MgF_2_) with different thicknesses of MAPI absorbers (220, 250, and 270 nm) measured under properly calibrated illumination conditions and (**b**) the corresponding EQE and 1– reflectance spectra. (**c**) *j–V* curve of the tandem cell with a 250-nm-thick perovskite layer scanned in both forward and reverse directions. (**d**) Normalized average efficiency of 6 tandem devices as a function of time while being stored in a desiccator (retained at 26 ~ 27 °C, ca. 24% relative humidity, and 0.1 atm)

Moreover, spectral mismatch in solar simulators can lead to inaccurate current matching in series-connected tandem solar cells because each subcell responds differently—depending on how the simulator is calibrated [55, 56]. To ensure accurate *j*–*V* measurements of monolithic tandem devices, we calibrated the light intensity of the solar simulator individually using Si reference cells with different window materials (KG5 or BK7). These were selected based on the current-limiting subcell. The limiting subcell was identified by extracting the *j*_SC, int_ from the EQE spectra (Fig. 6b). The EQE analysis revealed that the tandem device with a 250-nm-thick MAPI layer produced the most balanced current generation, with top and bottom subcells producing 15.7 and 15.9 mA cm^–2^, respectively. In contrast, the device with a thinner 220-nm-thick MAPI layer was limited by the top subcell and yielded only 14.8 mA cm^–2^. On the other hand, when the MAPI layer was increased to 270 nm, the perovskite top cell absorbed more light in the 600–800 nm range. However, optical interference induced significant reflection losses above the bandgap (800–1000 nm), which is confirmed by the 1– *R* (reflectance) curve. Consequently, the bottom cell received insufficient light, which decreased its current to 14.4 mA cm^–2^. Taking the current-limiting subcell into account, we calibrated the solar simulator accordingly for each tandem configuration and conducted *j*–*V* measurements (Fig. 6a). The resulting *j*_SC_ for devices with 220-nm, 250-nm, and 270-nm-thick MAPI layers were 14.9, 15.8, and 14.9 mA cm^–2^, respectively, which is in good agreement with the *j*_SC, int_ values obtained from the EQE measurements.

**Fig. 7:**
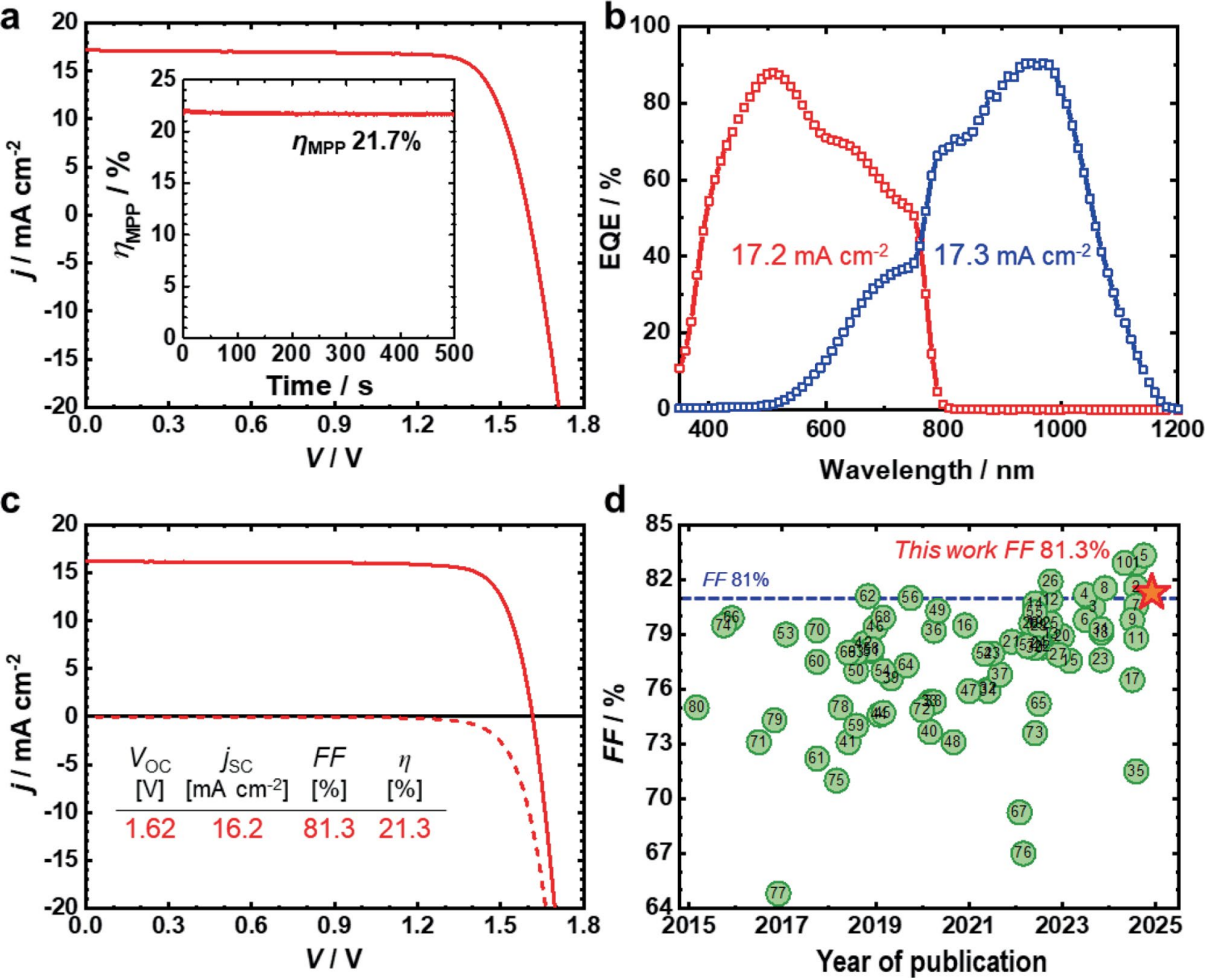
(**a**) *j*–*V* curve of the champion tandem device (Si/ITO-P/f-Cu:NiO_*x*_/MAPI/PCBM/ZnO-NP/IZO/Ag/MgF_2_) and (**b**) the corresponding EQE spectra. Inset of part a: Steady-state PCE measured using maximum-power-point tracking at *V*_MPP_ of 1.37 V. (**c**) *j − V* curve of an optimal tandem device (with a 250-nm-thick perovskite layer) exhibiting the highest *FF*. (**d**) Compilation of reported *FF* values for monolithic 2T perovskite/Si tandem solar cells (the numeral inside the symbols is the reference number in Table S3)

All PV parameters of the corresponding tandem devices are listed in Table 1, and statistical distributions are presented in Fig. S20. Figure 6c shows the *j*–*V* characteristic of the optimally current-matched tandem device (250-nm-thick MAPI) under both reverse and forward scans; they delivered PCEs of 20.4% and 20.6%, respectively, with negligible hysteresis and a high *FF* exceeding 80%. Notably, a maximum *FF* of 81.3% was achieved (Fig. 7c), placing it among the few reported values for monolithic perovskite/Si tandem devices to the best of our knowledge (Fig. 7d and Table S3). The hysteresis-less and high *FF* clearly indicates that the PEDOT:PSS-engineered RJ effectively minimizes electrical losses and contributes to both better performance and improved reliability. Furthermore, unencapsulated tandem devices incorporating the PEDOT:PSS-engineered RJ retained over 98% of their initial efficiency after prolonged storage in a desiccator, as shown in the normalized PCE trend over time (Fig. 6d). Figure 7a presents the *j*–*V* curve of the best-performing monolithic perovskite/Si tandem solar cell. The top and bottom cells were precisely aligned using a metal frame holder during integration, which effectively minimized shunting pathways and reduced current losses. It is noted that the tandem device exhibited a slight increase in efficiency after several days due to aging effects (Fig. S20) [57]. As a result, the stabilized device achieved a PCE of 21.95%, with a *V*_OC_ of 1.60 V, *j*_SC_ of 17.2 mA cm^–2^, and *FF* of 79.8%. The corresponding EQE spectra in Fig. 7b confirm the current matching of the two subcells, showing *j*_SC, int_ of 17.16 mA cm^–2^ and 17.28 mA cm^–2^ for the perovskite top and Si bottom cells, respectively, which is in close agreement with the *j*_SC_ obtained from the *j*–*V* curve, 17.19 mA cm^–2^. This tandem performance represents an 8.2% improvement over a standard Al-BSF Si SJ solar cell with an efficiency of 20.29% [58]. Given that a standalone *p*-type homojunction Si bottom cell with a 20-nm-thick ITO top electrode exhibits an efficiency of only 10.1% (Fig. S23a and Table S6), the monolithic integration with a perovskite top cell led to a remarkable relative increase (117.3%) in tandem efficiency. These results highlight the immense potential of tandem architectures and suggest that further efficiency gains are possible through device-structure optimization aimed at minimizing optical losses.

**Table 1:** PV parameters of the representative interface-engineered perovskite/Al-BSF Si tandem devices shown in Fig. 6a

Thickness of MAPI^a^	V_OC_ [V]	j_SC_ [mA cm^–2^]	FF [%]	PCE [%]
220 nm	1.53 (1.55 ± 0.03)^*b*^	14.9 (15.0 ± 0.3)	81.0 (77.0 ± 2.9)	18.4 (18.0 ± 0.7)
250 nm	1.62 (1.60 ± 0.03)	15.8 (16.3 ± 0.5)	80.3 (77.1 ± 2.2)	20.5 (20.1 ± 0.7)
270 nm	1.61 (1.59 ± 0.01)	14.9 (14.6 ± 0.8)	79.2 (78.3 ± 1.9)	19.0 (18.3 ± 1.0)

^*a*^ The solar simulator was calibrated using two Si reference cells with different optical filters: a Si reference with a KG5 filter was used for 220-nm and 250-nm MAPI devices, while a Si reference cell with a BK7 filter was used for the 270-nm MAPI device. ^*b*^ The values in parentheses are the average photovoltaic parameters calculated from 7, 13, and 10 devices for the 220, 250, and 270-nm MAPI devices, respectively

### Optical simulation-based perspective on perovskite/Al-BSF tandem design

**Fig. 8:**
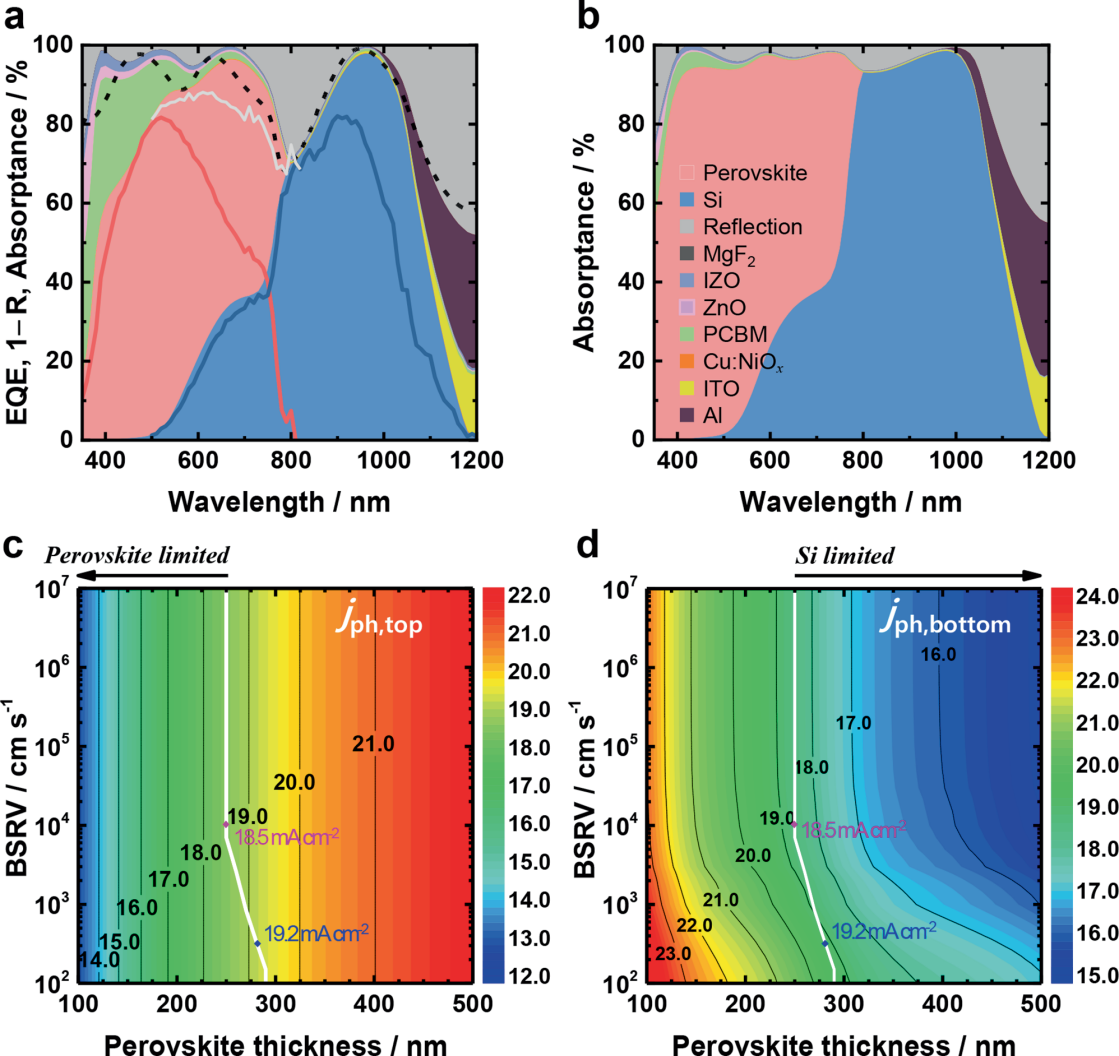
(**a**) Simulated optical absorptance and reflectance profiles for the representative tandem cell with 250-nm-thick MAPI shown in Fig. 6. The measured EQE of the perovskite top cell (solid red line) and the Si bottom cell (solid blue line) are plotted together with the measured total reflectance of the tandem cell (dashed black line). (**b**) Simulated optical absorptance profiles of the perovskite/Al-BSF Si tandem structure with an optimal thickness design to minimize parasitic absorption and reflection loss. Simulated *j*_ph_ of (**c**) perovskite and (**d**) Al-BSF Si subcells as functions of perovskite thickness and BSRV of Si, respectively. The white contour lines represent the matching current density of both subcells

Optical simulations were thus performed to provide a design guide for further optimization and to evaluate the performance improvement potential of our perovskite/Al-BSF Si tandem solar cells. The refractive indices (*n*) of most of the layers in the tandem architecture were determined using spectroscopic ellipsometry—with the exception of a few materials such as perovskite, Si, and Al, for which the *n* values were extracted from the literature [59, 60, 61, 62, 63]. The ray optics simulation software package (CROWM) was used to model light scattering at the back contact of the Si bottom cell. A detailed description of the simulation is provided in the Methods section. Figure 8a illustrates the simulated optical absorptance (*A*) of each layer for the tandem device fabricated in this study (Al/Si/ITO/Cu:NiO_*x*_/MAPI (250 nm)/PCBM/ZnO-NP/IZO/MgF_2_). Here, we first focus on the optical aspects before considering any adjustments due to the charge-collection characteristics of the subcells. The measured reflectance of the tandem cell was also co-plotted in Fig. 8a and found to agree reasonably well with the simulated one depicted by the dashed curve, thus supporting the validity of the present optical model.

It can be seen in Fig. 8a that there are three major causes of optical losses in the present perovskite/Al-BSF Si tandem cells, namely, the parasitic absorption loss by PCBM in the wavelength range of *λ*/nm < 600, the reflection losses in the range of 700 < *λ*/nm < 950, and that in the range of *λ*/nm > 1000. Among these, the suppression of the reflection at *λ*/nm > 1000 is limited with the current Al-BSF cell architecture without texturing. We thus carried out further simulations to find optimized optical designs that could potentially minimize surface reflectance while simultaneously reducing parasitic absorption by PCBM to a practically achievable level. The detailed calculation procedure with varying thicknesses of IZO, Cu:NiO_*x*_, and ITO can be found in the description associated with Figs. S23–S25. The simulated reflectance loss contour maps as functions of the IZO, Cu:NiO_*x*_, and ITO thicknesses, as shown in Fig. S24, reveal that the minimum reflectance can be achieved with a 30-nm-thick IZO top layer and a 10-nm-thick ITO interlayer. Figure 8b shows the absorptance profiles calculated according to this optimized optical design. One can observe that the reflectance loss— particularly in the spectral range of 700 < *λ*/nm < 950—is greatly suppressed compared to that depicted in Fig. 8a (see also Table S4 for the detailed simulation results). Thanks to this optical modification, the reflectance loss and the parasitic absorption loss by PCBM were minimized from 5.0 to 3.0 mA cm^–2^ and from 2.3 to 0.4 mA cm^–2^, respectively. As a consequence, the *j*_ph_ of the perovskite and Si subcells (which were adjusted according to Eq. S1 using the IQE evaluated with PC1D simulation) increased by 2.2 and 2.3 mA cm^–2^, respectively.

Finally, to provide prospects for the tandem cell efficiency achievable through state-of-the-art improvements in the BSRV of the Si bottom cell for the optimized IZO top and ITO interlayer thicknesses, we simulated the *j*_ph_ of subcells as functions of the BSRV of Si and perovskite thickness. The results are shown in Fig. 8c–d. The white contour lines represent the current matching conditions where the *j*_ph_ values of the perovskite and Si subcells are identical. Figure 8c–d show that, while the *j*_ph_ of the perovskite top cell depends only on its thickness, the *j*_ph_ of the Si bottom cell can be improved by decreasing the BSRV as well as the perovskite thickness. When the BSRV of the Si subcell is set to 10^4^ cm s^–1^, as determined by fitting the PC1D-simulated EQE to the measured spectrum (Fig. S23), the resulting matched current density is 18.5 mA cm^–2^. This already represents a significant improvement that can be achieved through the optimized optical design shown above. On the other hand, a state-of-the-art Al-BSF solar cell is characterized by a much stronger BSF, exhibiting ca. 300 cm s^–1^ of BSRV. The present simulations (as shown in Figs. 8c–d) suggest that the matching current can ultimately be improved (up to 19.2 mA cm^–2^) by employing Al-BSF solar cells with the state-of-the-art BSF quality and concurrently increasing the perovskite layer thickness to ca. 280 nm. By combining this current density with the highest *V*_OC_ and *FF* achieved in this work (1.63 V and 81.3%), the optimized perovskite/Al-BSF Si tandem solar cell is projected to reach a power conversion efficiency of 25.4%. This value surpasses the practical efficiency limit of commercial Al-BSF and PERC cells, which are estimated at 21.5% and 24.5% of PCE in the long term [64, 65].

## Conclusions

This work presents a robust interface engineering strategy based on a sacrificial layer approach to suppress the formation of a resistive SiO_*x*_ barrier layer, thereby enabling high-performance, monolithic integration of perovskite/Al-BSF Si tandem solar cells. A key advance is the use of an ultrathin PEDOT:PSS-engineered recombination junction, which enhances charge recombination by inducing strong interfacial electric fields and stepwise energy band alignment, as confirmed by band alignment analysis. The engineered RJ enabled monolithic perovskite/Si tandem solar cells with excellent reproducibility and outstanding performance, including a high fill factor of 81.3%. With optimized perovskite thickness for current matching, the champion device delivered a PCE of 21.95% under *j*–*V* measurement and 21.7% under MPP tracking. Optical simulations further predict that integrating a state-of-the-art Al-BSF Si bottom cell could push the tandem efficiency to 25.4%. Looking ahead, incorporating mainstream Si bottom cell technologies such as PERC could drive monolithic tandem efficiency even further and pave the way toward commercially viable, next-generation tandem photovoltaics.

## Electronic supplementary material

Below is the link to the electronic supplementary material.

Supplementary Material 1

## Data Availability

The manuscript includes data as electronic supplementary material. The corresponding authors can provide additional data upon reasonable request.
