# Correction: Sacrificial layer concept interface engineering for robust, lossless monolithic integration of perovskite/Si tandem solar cells yielding high fill factor of 0.813

**DOI:** 10.1186/s40580-025-00497-y

**Published:** 2025-06-30

**Authors:** Yoon Hee Jang, Youngseok Lee, Hyeon Sik Seo, Haram Lee, Kyoung-jin Lim, Jung-Kun Lee, Jaeyeong Heo, Inho Kim, Doh-Kwon Lee

**Affiliations:** 1 Advanced Photovoltaics Research Center, Korea Institute of Science and Technology (KIST), Seoul, 02792 Republic of Korea; 2 Center for Semiconductor Technology, Korea Institute of Science and Technology (KIST), Seoul, 02792 Republic of Korea; 3 PVCVD Team, R&D Center, Jusung Engineering Co., Ltd, Yongin, 17094 Republic of Korea; 4 Department of Mechanical Engineering and Materials Science, University of Pittsburgh, Pittsburgh, PA 15260 USA; 5 Department of Materials Science and Engineering, Optoelectronics Convergence Research Center, Chonnam National University, Gwangju, 61186 Republic of Korea; 6 Division of Nano and Information Technology, KIST School, University of Science and Technology, Seoul, 02792 Republic of Korea; 7 Present Address: Cell R&D Team, Hanhwa Q CELLS, Jincheon-Gun, Republic of Korea

**Correction to: Nano Convergence (2025) 12:24** 10.1186/s40580-025-00492-3

In the original publication of this article [1], there was an error in Fig. 1. The graphical abstract has been duplicated in the article PDF but appears correctly online for Fig. 1. The incorrect and correct Fig. 1 are shown in this correction article. The original article has been corrected.

Incorrect Figure 1:
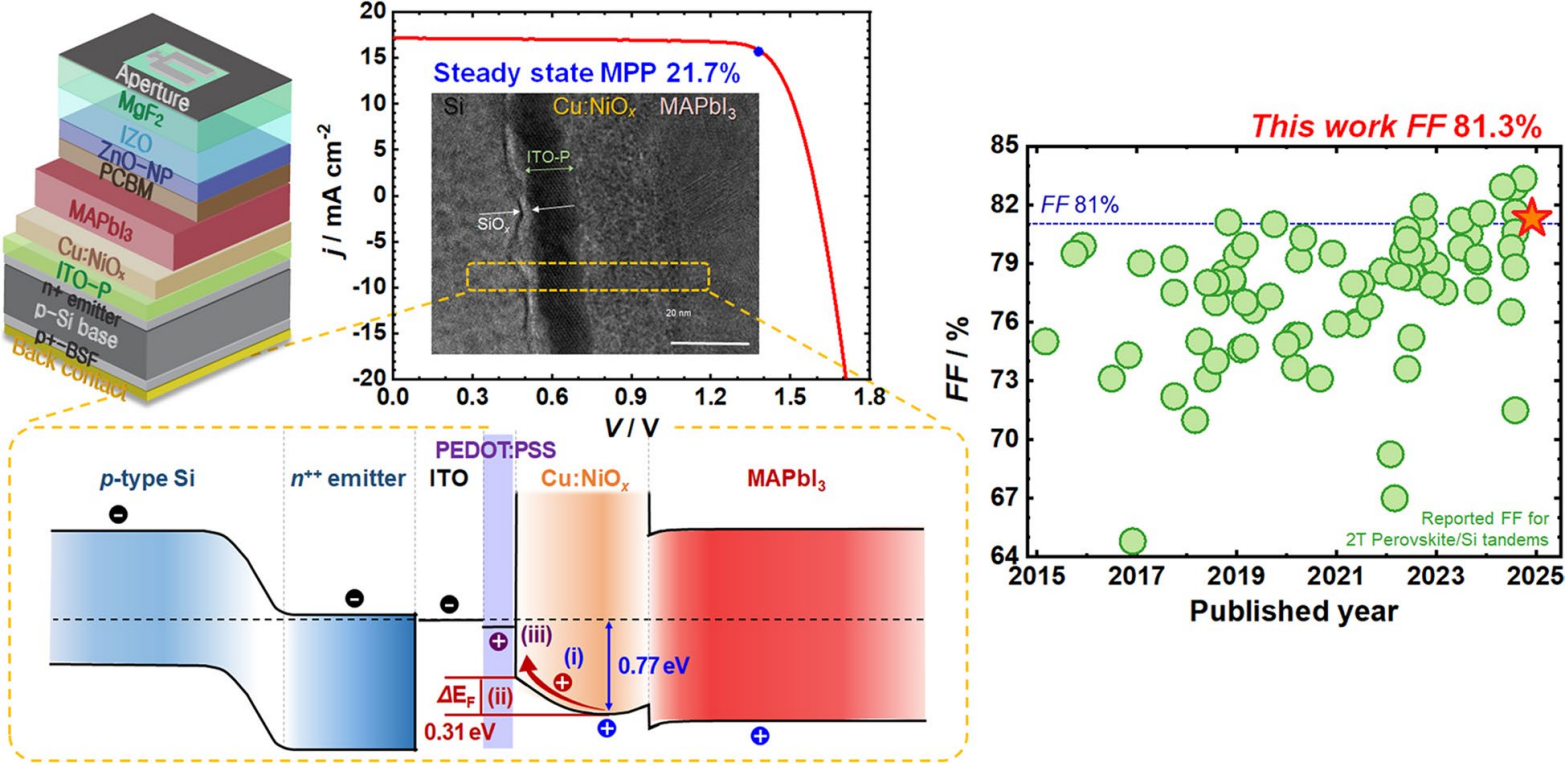


Correct Fig. 1:

**Fig. 1:**
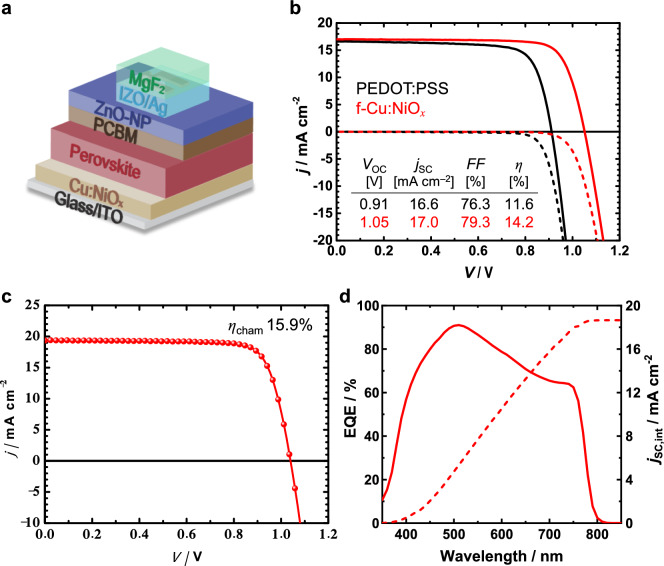
(**a**) Illustration of a semitransparent perovskite solar cell (ST-PSC) device with the structure glass/ITO/Cu:NiO_*x*_/MAPI/PCBM/ZnO-NP/IZO/Ag/MgF_2_. (**b**) *j*–*V* characteristics of representative ST-PSCs with different HTLs, PEDOT:PSS and f-Cu:NiO_*x*_ (without an antireflection (AR) layer). (**c**) *j*–*V* curve and (d) EQE spectrum of the best f-Cu:NiO_*x*_-based ST-PSC with an MgF_2_ AR layer. Note that all *j*–*V* and EQE curves were measured in substrate configuration
