# Supplementary material for: Sacrificial layer concept interface engineering for robust, lossless monolithic integration of perovskite/Si tandem solar cells yielding high fill factor of 0.813
Source: Nano Converg. 2025 May 27;12:24. doi: 10.1186/s40580-025-00492-3 (PMC12116411; doi:10.1186/s40580-025-00492-3)
Supplement: Supplementary file 1 — Supplementary Material 1 [file 40580_2025_492_MOESM1_ESM.docx]

Supporting Information

Sacrificial layer concept interface engineering for robust, lossless monolithic integration of perovskite/Si tandem solar cells yielding high fill factor of 0.813

Yoon Hee Jang^1, †^, Youngseok Lee^2, †^, Hyeon Sik Seo^2^, Haram Lee^1^, Kyoung-jin Lim^3^, Jung-Kun Lee^4^, Jaeyeong Heo^5, *^, Inho Kim^2, 6, *^, and Doh-Kwon Lee^1, 6, *^

^1^ *Advanced Photovoltaic Research Center, Korea Institute of Science and Technology (KIST), Seoul 02792, Republic of Korea*

^2^ *Center for Semiconductor Technology, Korea Institute of Science and Technology (KIST), Seoul 02792, Republic of Korea*

^3^ *PVCVD Team, R&D Center, Jusung Engineering Co., Ltd., Yongin 17094, Republic of Korea*

^4^ *Department of Mechanical Engineering and Materials Science, University of Pittsburgh, PA 15260, USA*

^5^ *Department of Materials Science and Engineering, Optoelectronics Convergence Research Center, Chonnam National University, Gwangju 61186, Republic of Korea*

^6^ *Division of Nano and Information Technology, KIST School, Korea University of Science and Technology, Seoul 02792, Republic of Korea*

**^*^ Corresponding authors.** E-mail addresses: dklee@kist.re.kr (D.-K. Lee), inhok@kist.re.kr (I. Kim), jheo@jnu.ac.kr (J. Heo)

^†^ Y. H. J. and Y. L contributed equally to this work.

**Contents:**

Number of pages: 47

Number of figures: 25

Number of tables: 6

**Fig. S1.** Power conversion efficiency evolution of published perovskite/Si monolithic tandem solar cells. Structures of homojunction Si bottom cells were specified in the figure.

**Fig. S2.** Optimization of optical and electrical properties of ITO thin films: (a) Transmittance spectra and (b) resistivity of 200-nm-thick ITO films sputtered with various oxygen mixing ratios (${X_{O}}_{2}$) during the deposition, ranging from 0 to 5%. (c) Haacke figure of merit (FOM) equation [1], where *T, ρ*, and *d* denote the transmittance, resistivity, and thickness of ITO film, respectively. (d) Summary of optical and electrical properties of the deposited ITO films.

**Fig. S3.** (a) Top-view and (b) cross-sectional FE-SEM images of Cu:NiO*_x_* films prepared by the control method (annealing at 300 °C and slow cooling, denoted as c-Cu:NiO*_x_* (coarse)) and the optimized method (including pre-annealing at 100 °C and fast quenching, denoted as f-Cu:NiO*_x_* (fine)).

**Fig. S4.** Effects of Cu doping into NiO*_x_* on the perovskite (MAPbI_3_; MAPI) film and solar cell device: (a) Device structure of Cu:NiO*_x_* HTL-based opaque PSC. (b) Top-view FE-SEM images of (b) c-NiO*_x_* and c-Cu:NiO*_x_* films and (c) MAPI films prepared on different HTLs (c-NiO*_x_* and c-Cu:NiO*_x_*). (d) *j*–*V* curves of c-NiO*_x_* and c-Cu:NiO*_x_* HTL-based opaque devices.

**Fig. S5.** Histograms of semitransparent perovskite solar cells (ITO/HTL/MAPI/PCBM/ZnO-NP/IZO) with different HTLs, PEDOT:PSS (black) and f-Cu:NiO*_x_* (red) for (a) *V*_OC_, (b) *j*_SC_, (c) *FF*, and (d) PCE. The number of devices used to calculate the statistics for PEDOT:PSS and Cu:NiO*_x_* HTLs is 24 and 32, respectively.

**Fig. S6**. Top-view FE-SEM images of MAPI films prepared through the identical procedure on different HTLs, PEDOT:PSS and c-Cu:NiO*_x_*.

**Fig. S7.** Normalized PCE with standard deviation of ST-PSCs employing different HTLs as a function of storage time under inert condition. The statistics were calculated with 5 cells with PEDOT:PSS and 4 cells with f-Cu:NiO*_x_*, respectively.

**Table S1.** Summary of PV parameters for NiO*_x_*-based semitransparent *p-i-n* configuration perovskite solar cells.

| **Device structure** | **Configuration** | ***V*_OC_**  **[V]** | ***j*_SC_**  **[mA cm^-2^]** | ***FF***  **[%]** | ***η***  **[%]** | **Published year** | **Ref.** |
| --- | --- | --- | --- | --- | --- | --- | --- |
| **ITO/Cu:NiO*_x_*/MAPbI_3_/PC_60_BM/ZnO-NP/IZO/MgF_2_**  **(w/o MgF_2_ layer)** | **Substrate** | **1.04** | **19.4** | **79.0** | **15.9**  **(14.2)** | ***This work*** |  |
| ITO/**NiO*_x_***/Cs_0.05_(FA_0.84_MA_0.16_)_0.95_Pb(I_0.75_Br_0.25_)_3_/LiF/C_60_/SnO_2_/IZO/MgF_2_ |  | 1.27 | 19.7 | 77.8 | 19.4 | 2024 | [2] |
| ITO/**NiO*_x_***/2PACz+MeO-2PACz/FAMAPb(IBr)_3_/C_60_/ALD-SnO_2_/IZO/Ag |  | 1.17 | 20.3 | 80.7 | 19.2 | 2023 | [3] |
| ITO/**NiO*_x_***/Cs_0.17_FA_0.83_Pb(Br_0.17_I_0.83_)_3_/LiF/C_60_/SnO_2_/ZTO/ITO/LiF |  | 0.98 | 18.7 | 79.0 | 14.5 | 2018 | [4] |
| ITO/**NiO*_x_***/Cs_0.17_FA_0.83_Pb(Br_0.17_I_0.83_)_3_/LiF/PC_60_BM/SnO_2_/ZTO/ITO/LiF |  | 0.98 | 18.7 | 78.8 | 14.5 | 2017^]^ | [5] |
| ITO/**NiO-NPs**/Cs_0.05_(MA,FA)_0.95_Pb(I_0.9_Br_0.1_)_3_/PC_60_BM/ZnO-NP/ALD-ZnO/ITO/MgF_2_ |  | 1.00 | 20.4 | 70.3 | 14.4 | 2018 | [6] |
| ITO/**NiO**/MAPbI_3_/PCBM/ZnO/ITO/Ag |  | 0.98 | 18.9 | 77.0 | 14.3 | 2019 | [7] |
| ITO/**NiO*_x_***/Me-4PACz/Rb_0.05_Cs_0.05_[(FA_0.83_MA_0.17_)]_0.9_Pb(I_0.83_Br_0.17_)_3_/MDADI/C_60_/SnO_2_/ITO/LiF | Superstrate | 1.24 | 21.5 | 81.9 | 21.8 | 2023 | [8] |
| ITO/**NiO_x_**/FA_0.87_Cs_0.13_Pb(I_0.87_Br_0.13_)_3_/PCBM/BCP/IO:GT |  | 1.12 | 19.3 | 82.9 | 17.9 | 2022 | [9] |
| MgF_2_/ITO/**NiO-NPs**/PTAA/Cs_0.05_(MA_0.17_FA_0.83_)_0.95_Pb(I_0.9_Br_0.1_)_3_/PCBM/ZnO-NP/ALD-ZnO/ITO/MgF_2_ |  | 1.07 | 19.4 | 78.1 | 16.3 | 2018 | [10] |
| ITO/NiO*_x_*/CsFAMA/C_60_/BCP/IZO/Ag |  | 1.00 | 21.5 | 75.5 | 16.2 | 2021 | [11] |
| ITO/NiO*_x_*/Cs_0.175_FA_0.75_MA_0.075_Pb(I_0.875_Br_0.125_)_3_/PMMA:PCBM/ZnO/IZTO/Ag |  | 1.07 | 19.0 | 76.9 | 15.7 | 2021 | [12] |
| ITO/**NiO*_x_***/FA_0.83_Cs_0.17_Pb(I_0.83_Br_0.17_)_3_/PCBM/SnO_2_/ITO |  | 0.97 | 20.3 | 79.0 | 15.7 | 2016 | [13] |
| ITO/**NiO*_x_***/Cs_0.175_FA_0.75_MA_0.075_Pb(I_0.875_Br_0.125_)_3_/PMMA:PCBM/ZnO/IGTO |  | 1.02 | 20.4 | 75.4 | 15.6 | 2021 | [14] |
| ITO/**NiO*_x_***/2PACz/Cs_0.1_(MA_0.17_FA_0.83_)_0.9_Pb(I_0.83_Br_0.17_)_3_/C_60_/SnO_2_/ITO/PEN foil |  | 1.1 | 18.7 | 74.7 | 15.4 | 2022 | [15] |
| ITO/**NiO*_x_***/CsPbI_2_Br/Ti_0.9_Sn_0.1_O_2_/IZO/MgF_2_ |  | 1.25 | 15.29 | 79.27 | 15.4 | 2023 | [16] |
| ITO/**NiO*_x_***/MAPbI_3_/PC_61_BM/ZrAcac/PEI/Ag(9 nm)/Ta_2_O_5_(25 nm) |  | 1.09 | 18.8 | 73.6 | 15.1 | 2019 | [17] |
| IZRO/**NiO*_x_***/MAPbI_3_/PC_60_BM/ZnO-NP/BCP/IZRO |  | 1.04 | 19.1 | 76.0 | 15.1 | 2019 | [18] |
| ITO/**NiO*_x_***/MAPbI_3_/PC_60_BM/ZnO-NP/DPO^a^/ITO |  | 1.07 | 19.1 | 73.6 | 15 | 2019 | [19] |
| ITO/**NiO*_x_***/DEA/MAPbI_3_/C_60_(CH_2_)(lnd)/PN_4_N/Ag(14 nm) |  | 1.13 | 14.7 | 76.0 | 12.6 | 2017 | [20] |
| ITO/**NiO*_x_***/MAPbI_3_/PC_61_BM/AZO/ITO |  | 1.06 | 19.7 | 60.0 | 12.5 | 2019 | [21] |

^a^(2-(1,10-phenanthrolin-3-yl)naphth-6-yl)diphenylphosphine oxide

**Fig. S8.** *j*–*V* characteristics of monolithic MAPI/Al-BSF Si tandem devices that have ITO/c-Cu:NiO*_x_* recombination contacts for various ITO thicknesses of (a) 5 nm, (b) 10 nm, and (c) 20 nm.

**Fig. S9.** *j*–*V* characteristics of (a) MaPbI_3_/CuInSe_2_ and (b) MaPbI_3_/Al-BSF Si monolithic tandem solar cells with TCO/PEDOT:PSS recombination junctions.

**Fig. S10.** Transmittance spectra of f-Cu:NiO*_x_* and PEDOT:PSS/f-Cu:NiO*_x_* films prepared on ITO substrate.

**Fig. S11.** (a,b) *j*–*V* curves and (c) statistical distribution of PV parameters for ST-PSC devices incorporating (a) c-Cu:NiO*_x_* and (b) f-Cu:NiO*_x_*, with and without the introduction of the PEDOT:PSS sacrificial layer.

**Fig. S12.** PV performance of perovskite/Al-BSF Si monolithic tandem devices with different RJs: ITO-P/c-Cu:NiO*_x_* (in red) and ITO/c-Cu:NiO*_x_* (in blue). (a) Representative *j*–*V* curves and (b) statistic distribution of PV parameters for both sets of 10 devices.

**Fig. S13.** (a) XPS elemental depth profiles of Si/ITO-P/f-Cu:NiO*_x_* and Si/ITO/f-Cu:NiO*_x_*. High resolution Si 2p spectra as a function of sputter etching time collected from (b) Si/ ITO-P/f-Cu:NiO*_x_* and (c) Si/ITO/f-Cu:NiO*_x_*, respectively. Note that the shaded areas in part a indicate the time at which the signal corresponding to SiO*_x_* (~102.5 eV) appears in part b and c to the time at which it disappears.

**Fig. S14.** HAADF-STEM and elemental mapping images of recombination junctions: (a) Si(*n*^++^)/ITO-P/c-Cu:NiO*_x_* and (b) Si(*n*^++^)/ITO/c-Cu:NiO*_x_*. (c,d) Enlarged views for Sn and O distributions marked on parts a and b. The circled areas indicate that the oxygen signal is found further down compared to the Sn signal, indicating the presence of another oxide, highly likely SiO*_x_*, beneath the ITO.

**Fig. S15.** (a,b) STEM images and (c,d) EDS line mapping for recombination junctions: (a,c) ITO-P/c-Cu:NiO*_x_* and (b,d) ITO/c-Cu:NiO*_x_*. (e) Enlarged EDS line profiles for sulfur along with the fitted curves with a Gaussian function.

**Fig. S16.** (a) Device structures and (b) typical *j*–*V* curves of single-junction semitransparent perovskite solar cells without and with PEDOT:PSS interlayer fabricated on phosphor-diffused *n*-type Si wafer substrates.

**Fig. S17.** UPS analysis to determine the band alignment near the RJ of Al-BSF Si/MAPI tandems: Secondary electron cut-off region (left) and valence band edge region (right) of He I (21.2 eV) UPS spectra of various configurations of samples: (a) ITO, (b) ITO/PEDOT:PSS, (c) ITO/f-Cu:NiO*_x_*, and (d) ITO/PEDOT:PSS/f-Cu:NiO*_x_*. The evaluated energy levels for work function (*Φ*) and valance band (VB) edge were denoted in the figures.

**Fig. S18.** Electronic energy band diagram of entire monolithic Al-BSF Si/MAPbI_3_ tandem solar cells with different recombination junctions: (a) ITO/f-Cu:NiO*_x_* (control) and (b) ITO-P/f-Cu:NiO*_x_* (modified).

**Fig. S19.** (a) Cross-sectional FE-SEM images, (b) *j*–*V* curves, and (c) EQE and transmittance (*T*) spectra of semitransparent perovskite devices with different thicknesses of perovskite absorber, where the *j*–*V*, EQE, and transmittance curves were measured in substrate configuration (without MgF_2_ layer). (d) [*hν* ln(1−EQE)]^2^ *vs*. *hν* curves near the band-edge regime extracted from the EQE curves. The bandgap value evaluated therefrom was 1.60 ± 0.01 eV for all perovskite devices irrespective of the absorber thickness.

**Table S2.** PV parameters for semitransparent *p-i-n* configuration perovskite solar cells (ITO/f-Cu:NiO*_x_*/MAPI/PCBM/ZnO-NP/IZO/Ag) with different thicknesses of MAPI layer as shown in **Figure S19**b.

| Thickness of MAPI | *V*_OC_  [V] | *j*_SC_  [mA cm^-2^] | *FF*  [%] | *η*  [%] |
| --- | --- | --- | --- | --- |
| 220 nm | 1.00  (1.00 ± 0.01)^a^ | 14.8  (14.7 ± 0.2) | 80.1  (76.8± 1.9) | 11.9  (11.3 ± 0.4) |
| 250 nm | 1.05  (1.01 ± 0.02) | 16.6  (15.5 ± 0.7) | 79.0  (76.4 ± 2.1) | 13.8  (11.9 ± 0.7) |
| 270 nm | 1.05  (1.01 ± 0.02) | 17.0  (16.4 ± 0.5) | 79.3  (76.9 ± 2.6) | 14.2  (12.8 ± 0.8) |

^a^Parentheses are average of PV parameters for each absorber thickness. The number of devices used to calculate the statistics for 220, 250, and 270 nm of MAPI are 12, 70, and 41, respectively.

 **Fig. S20.** Statistics of PV parameters of PEDOT:PSS-modified perovskite/Al-BSF Si monolithic tandem devices (Si/ITO-P/f-Cu:NiO*_x_*/MAPI/PCBM/ZnO-NP/IZO/Ag/ MgF_2_) with different thicknesses of MAPI absorbers (220, 250, and 270 nm).

**Table S3.** Comparison of PV parameters for perovskite/Si monolithic tandem solar cells with an emphasis on the *FF* value.

| *No*. | Si  Bottom cell | Perovskite Top cell | *V*_OC_  [V] | *j*_SC_  [mA cm^-2^] | ***FF***  **[%]** | *η*  [%] | *η_MPP_*  [%] | Area  [cm^2^] | *Published year* | *Ref.* |
| --- | --- | --- | --- | --- | --- | --- | --- | --- | --- | --- |
|  | ***p*-type Homojunction (Al-BSF)** | ***p-i-n*** | **1.62** | **16.2** | **81.3** | **21.3** | **-** | **0.2637** | **This work^a^** |  |
| 1 | SHJ | *p-i-n* | 1.98 | 20.7 | **82.9** | 34.0 | 34.0 | 1.004 | 2024 | [22] |
| 2 | SHJ | *p-i-n* | 1.99 | 21.0 | **81.6** | 34.0 | - | 1.04 | 2024 | [23] |
| 3 | SHJ | *p-i-n* | 1.95 | 20.9 | **80.5** | 32.8 | 32.5 | 1.03 | 2023 | [24] |
| 4 | SHJ | *p-i-n* | 1.98 | 20.2 | **81.2** | 32.5 | - | 1 | 2023 | [25] |
| 5 | SHJ | *p-i-n* | 1.87 | 20.7 | **83.3** | 32.1 | 31.9 | 1.015 | 2024 | [26] |
| 6 | SHJ | *p-i-n* | 1.91 | 20.5 | **79.8** | 31.3 | - | 1.1677 | 2023 | [27] |
| 7 | SHJ | *p-i-n* | 1.89 | 20.5 | **80.6** | 31.4 | 31.1 | 1 | 2024 | [28] |
| 8 | *n*-type Homojunction  (TOPCon) | *p-i-n* | 1.93 | 19.6 | **81.5** | 30.8 | 30.7 | 1.087 | 2023 | [2] |
| 9 | SHJ | *p-i-n* | 1.90 | 20.1 | **79.8** | 30.5 | 30.3 | 1 | 2024 | [29] |
| 10 | SHJ | *p-i-n* | 1.81 | 20.0 | **82.9** | 30.1 | 29.4 | 0.00875 | 2024 | [30] |
| 11 | PERX/TOPCon  (Q.ANTUM) | *p-i-n* | 1.93 | 19.7 | **78.8** | 30.1 | - | 1.1 | 2024 | [31] |
| 12 | SHJ | *p-i-n* | 1.90 | 19.5 | **80.9** | 29.8 | 29.8 | 1 | 2022 | [32] |
| 13 | SHJ | *p-i-n* | 1.89 | 19.8 | **79.0** | 29.6 | 29.4 | 1 | 2022 | [33] |
| 14 | SHJ | *p-i-n* | 1.91 | 19.8 | **80.7** | 29.3 | 29.3 | 1 | 2022 | [34] |
| 15 | SHJ | *p-i-n* | 1.93 | 19.5 | **77.6** | 29.1 | 29.2 | 0.15 | 2023 | [35] |
| 16 | SHJ | *p-i-n* | 1.90 | 19.3 | **79.5** | 29.1 | 29.2 | 1.064 | 2020 | [36] |
| 17 | SHJ | *p-i-n* | 1.88 | 20.2 | **76.5** | 29.0 | 28.5 | 1 | 2024 | [37 |
| 18 | SHJ | *p-i-n* | 1.91 | 19.1 | **79.1** | 28.9 | - | 1 | 2023 | [38] |
| 19 | SHJ | *p-i-n* | 1.86 | 19.6 | **79.6** | 28.9 | - | 1 | 2022 | [39] |
| 20 | SHJ | *p-i-n* | 1.85 | 20.1 | **78.9** | 28.9 | 28.6 | 1.05 | 2023 | [2] |
| 21 | SHJ | *p-i-n* | 1.87 | 19.6 | **78.6** | 28.9 | 28.6 | 1.03 | 2021 | [40] |
| 22 | PERC/TOPCon  (Q.ANTUM) | *p-i-n* | 1.91 | 19.3 | **78.3** | 28.8 | 28.7 | 1.006 | 2022 | [41] |
| 23 | SHJ | *p-i-n* | 1.85 | 20.1 | **77.6** | 28.8 | 28.3 | 1.05 | 2023 | [42] |
| 24 | SHJ | *p-i-n* | 1.92 | 19.0 | **78.5** | 28.6 | 28.5 | 1 | 2022 | [43] |
| 25 | SHJ | *p-i-n* | 1.85 | 19.4 | **19.6** | 28.5 | 28.2 | 0.5036 | 2022 | [44] |
| 26 | *n*-type Homojunction  (TOPCon) | *p-i-n* | 1.80 | 19.3 | **81.9** | 28.5 | 28.2 | 0.124 | 2022 | [45] |
| 27 | SHJ | *p-i-n* | 1.85 | 19.7 | **77.9** | 28.4 | 28.0 | 0.9767 | 2022 | [46] |
| 28 | SHJ | *p-i-n* | 1.79 | 19.5 | **79.6** | 27.8 | - | 1 | 2022 | [47] |
| 29 | SHJ | *p-i-n* | 1.83 | 19.0 | **79.5** | 27.6 | 27.3 | 0.5003 | 2022 | [48] |
| 30 | *n*-type Homojunction (TOPCon) | *p-i-n* | 1.79 | 19.7 | **78.3** | 27.6 | - | 1 | 2022 | [49] |
| 31 | SHJ | *p-i-n* | 1.82 | 19.0 | **79.3** | 27.4 | - | 0.25 | 2023 | [50] |
| 32 | SHJ | *p-i-n* | 1.84 | 19.6 | **76.0** | 27.4 | 27.4 | 1.03 | 2021 | [51] |
| 33 | SHJ | *p-i-n* | 1.89 | 19.1 | **75.3** | 27.1 | 27.0 | 1 | 2020 | [52] |
| 34 | SHJ | *n-i-p* | 1.83 | 19.5 | **75.9** | 27.1 | 27.0 | 1.03 | 2021 | [53] |
| 35 | SHJ | *n-i-p* | 1.91 | 19.8 | **71.5** | 27 | 27 | 1 | 2024 | [54] |
| 36 | SHJ | *p-i-n* | 1.76 | 19.2 | **79.2** | 26.7 | 26.5 | 1.001 | 2020 | [55] |
| 37 | SHJ | *p-i-n* | 1.78 | 19.2 | **76.8** | 26.2 | - | 1.03 | 2021 | [56] |
| 38 | SHJ | *p-i-n* | 1.82 | 19.2 | **75.3** | 26.2 | 26.1 | - | 2020 | [57] |
| 39 | SHJ | *p-i-n* | 1.77 | 19.2 | **76.6** | 26.1 | 26.0 | 0.7709 | 2019 | [58] |
| 40 | SHJ | *p-i-n* | 1.79 | 15.9 | **73.7** | 25.7 | 25.7 | 0.832 | 2020 | [59] |
| 41 | SHJ | *p-i-n* | 1.79 | 19.5 | **73.1** | 25.5 | 25.2 | 1.42 | 2018 | [60] |
| 42 | SHJ | *p-i-n* | 1.76 | 18.5 | **78.5** | 25.5 | - | 0.77 | 2018 | [61] |
| 43 | SHJ | *n-i-p* | 1.92 | 17.0 | **78.0** | 25.4 | - | 0.5003 | 2021 | [62] |
| 44 | SHJ | *p-i-n* | 1.79 | 19.0 | **74.6** | 25.4 | 25.2 | 1.1 | 2019 | [63] |
| 45 | *p*-type Nanocrystalline (Fz wafer) | *p-i-n* | 1.74 | 19.5 | **74.7** | 25.4 | 25.1 | 1.42 | 2019 | [64] |
| 46 | SHJ | *p-i-n* | 1.8 | 17.8 | **79.4** | 25.4 | - | 0.4225 | 2019 | [65] |
| 47 | SHJ | *p-i-n* | 1.8 | 18.5 | **75.9** | 25.2 | - | 0.832 | 2021 | [66] |
| 48 | SHJ | *p-i-n* | 1.73 | 19.8 | **73.1** | 25.1 | 25.0 | 0.8317 | 2020 | [67] |
| 49 | SHJ | *p-i-n* | 1.77 | 17.7 | **80.3** | 25.1 | 25.1 | 0.25 | 2020 | [68] |
| 50 | SHJ | *p-i-n* | 1.77 | 18.4 | **77.0** | 25.0 | - | 1 | 2018 | [69] |
| 51 | *n*-type Homojunction  (TOPCon) | *n-i-p* | 1763 | 17.8 | **78.1** | 24.5 | 24.1 | 1 | 2018 | [70] |
| 52 | SHJ | *p-i-n* | 1.68 | 18.6 | **77.9** | 24.4 | 24.6 | 1.008 | 2021 | [71] |
| 53 | SHJ | *p-i-n* | 1.65 | 18.1 | **79.0** | 23.6 | 23.6 | 1 | 2017 | [5] |
| 54 | SHJ | *p-i-n* | 1.67 | 18.3 | **77.0** | 23.5 | 23.4 | 0.1875 | 2019 | [7] |
| 55 | *p*-type Homojunction (Al-BSF) | *p-i-n* | 1.75 | 16.7 | **80.2** | 23.5 | 23.5 | 0.25 | 2022 | [72] |
| 56 | *n*-type Homojunction (PERC) | *n-i-p* | 1.73 | 16.5 | **81.0** | 23.1 | 23 | 4 | 2019 | [73] |
| 57 | SHJ | *n-i-p* | 2.04 | 14.3 | **78.4** | 23.0 | 22.0 | 0.5003 | 2022 | [74] |
| 58 | *n-*type Homojunction (Fz-wafer) | *n-i-p* | 1.70 | 17.2 | **78.2** | 22.9 | - | 1 | 2018 | [70] |
| 59 | SHJ | *n-i-p* | 1.78 | 17.1 | **74.0** | 22.8 | - | 0.13 | 2018 | [75] |
| 60 | SHJ | *n-i-p* | 1.75 | 16.8 | **77.5** | 22.8 | 22.0 | 0.25 | 2017 | [76] |
| 61 | *n*-type Homojunction (PERT) | *n-i-p* | 1.75 | 17.6 | **72.2** | 22.3 | 22.5 | 1 | 2017 | [77] |
| 62 | SHJ | *n-i-p* | 1.66 | 16.5 | **81.1** | 22.2 | 20.6 | 0.06 | 2018 | [78] |
| 63 | *n*-type Homojunction (PERC) | *n-i-p* | 1.74 | 16.2 | **78.0** | 21.9 | 21.8 | 16 | 2018 | [79] |
| 64 | SHJ | *n-i-p* | 1.84 | 15.2 | **77.3** | 21.6 | 21.6 | 0.25 | 2020 | [80] |
| 65 | SHJ | *n-i-p* | 1.81 | 15.7 | **75.2** | 21.4 | - | 0.25 | 2022 | [81] |
| 66 | SHJ | *n-i-p* | 1.69 | 15.8 | **79.9** | 21.4 | 21.2 | 0.17 | 2016 | [82] |
| 67 | *p*-type Homojunction (PERC/POLO) | *p-i-n* | 1.80 | 17.1 | **69.3** | 21.3 | - | 1 | 2022 | [83] |
| 68 | *p-type Homojunction (Al-BSF)* | *p-i-n* | 1.65 | 16.1 | **79.9** | 21.2 | 21.1 | 0.26772 | 2019 | [84] |
| 69 | *n*-type Homojunction (PERT) | *n-i-p* | 1.68 | 16.1 | **78.0** | 21.0 | 20.5 | 4 | 2018 | [85] |
| 70 | SHJ | *n-i-p* | 1.70 | 15.3 | **79.2** | 20.6 | 18.0 | 0.03 | 2017 | [86] |
| 71 | SHJ | *n-i-p* | 1.72 | 16.4 | **73.1** | 20.6 | 20.5 | 1.43 | 2016 | [87] |
| 72 | SHJ | *n-i-p* | 1.67 | 16.3 | **74.9** | 20.4 | - | 0.66 | 2020 | [88] |
| 73 | SHJ | *p-i-n* | 1.75 | 15.5 | **73.6** | 20.0 | 19.3 | 0.14 | 2022 | [15] |
| 74 | SHJ | *n-i-p* | 1.79 | 14.0 | **79.5** | 19.9 | 18.1 | 0.16 | 2016 | [89] |
| 75 | SHJ | *n-i-p* | 1.71 | 15.5 | **71.0** | 18.8 | - | 0.13 | 2018 | [90] |
| 76 | *n*-type Homojunction (TOPCon) | *n-i-p* | 1.78 | 14.4 | **67.0** | 17.3 | - | 25 | 2022 | [91] |
| 77 | *n*-type Homojunction (Al-BSF) | *n-i-p* | 1.64 | 15.3 | **64.8** | 16.3 | 16.0 | 1.43 | 2016 | [92] |
| 78 | *p-type Homojunction (Al-BSF)* | *p-i-n* | 1.43 | 15.3 | **75.0** | 16.2 | 16.0 | 1 | 2018 | [4] |
| 79 | SHJ | *n-i-p* | 1.62 | 13.0 | **74.3** | 15.7 | - | 0.17 | 2016 | [93] |
| 80 | *n*-type Homojunction (Al-BSF) | *n-i-p* | 1.58 | 11.5 | **75.0** | 13.7 | 13.7 | 1 | 2015 | [94] |

^a^PV parameters of a monolithic tandem device with the highest FF in this work are presented.

**Supplementary note: Optical simulation**

Equivalent current density (*j*_ph_) was evaluated by converting the absorptance of each layer into electricity as follows:

$j_{\mathrm{ph}}=\frac{q\lambda}{hc}\int\mathrm{IQE}\left( \lambda\right)\cdot A\left( \lambda\right)\cdot I_{\mathrm{solar}}\left( \lambda\right)d\lambda$ (S1)

where *q*, *h*, *λ*, *c*, IQE, *A*, and *I*_solar_ denote the elementary charge, Planck constant, wavelength and speed of light, internal quantum efficiency and absorptance of each layer, and the intensity of solar irradiation under a standard condition of AM 1.5G at 100 mW cm^–2^, respectively. The absorptances of each layer in tandem solar cells were calculated by using the commercial software package (CROWM). For the tandem cell structure, refer to **Fig. 2**a. The light scattering at the rear side of the Al-BSF bottom cells was considered by importing the AFM image into the optical simulation (**Fig. S21**). The refractive indices of each layer for the simulations except perovskite, Si and Al were determined by spectroscopic ellipsometry and presented in **Fig. S22**. For a given *Abs*, a maximum *j*_ph_ is obtained when there is no collection loss, *i.e.* the IQE is unity. For a given absorptance (*A*), a maximum *j*_ph_ is obtained when there is no collection loss, *i.e.* the IQE is unity. As shown in the breakdown analysis of optical absorptances (**Table S4**), the simulated *j*_ph_ value of the perovskite layer (16.3 mA cm^–2^) turned out to be in reasonable agreement with the measured *j*_SC,int_ (15.7 mA cm^–2^) (**Fig. 6**b), whereas that of the Si layer (19.8 mA cm^–2^) is much higher than the *j*_SC,int_ (15.9 mA cm^–2^) (**Fig. 6**b). The discrepancy is presumably attributed to the recombination losses that occur primarily at the metallized contact on the rear side of the Si bottom cell. For Al-BSF solar cells, the rear side recombination is known as one of the most significant limiting factors for device performance, and hence the formation of Al-BSF by Si-Al alloying is considered an crucial process for producing high-efficiency devices. Depending on the qualities of the Al-BSF such as the thickness and interfacial morphology, the recombination rate at the back contack, *i.e.*, back surface recombination velocity (BSRV), is known to be in the range of 300 to 10,000 cm s^–1^ [95–97]. The parameters employed in the PC1D simulations are provided in **Table S5**.

**
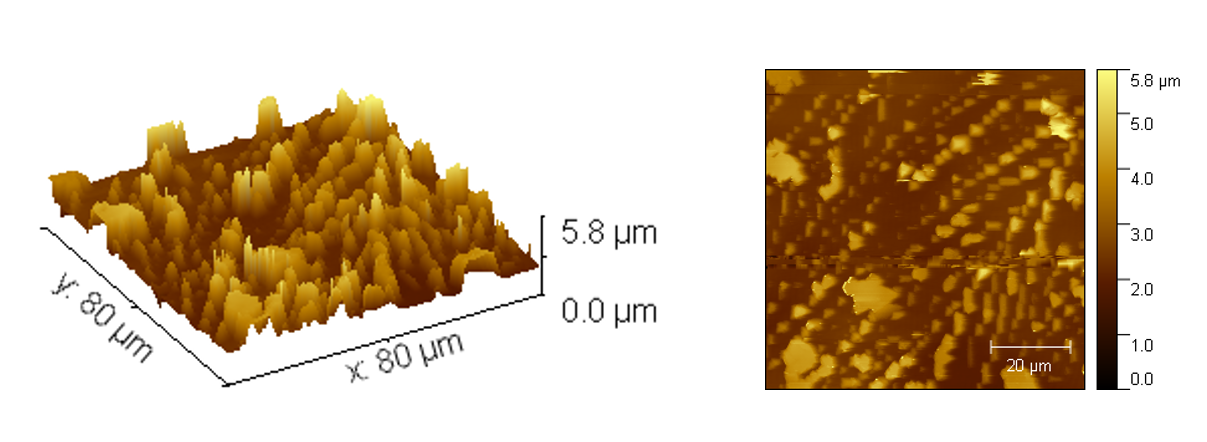
**

**Fig. S21.** AFM height images (3D and 2D on the left and right, respectively) of the rear side of the Al-BSF Si cell. The Al electrode on the rear side was chemically etched for the precise acquisition of the Si/Al interface morphology after the co-diffusion process.

**
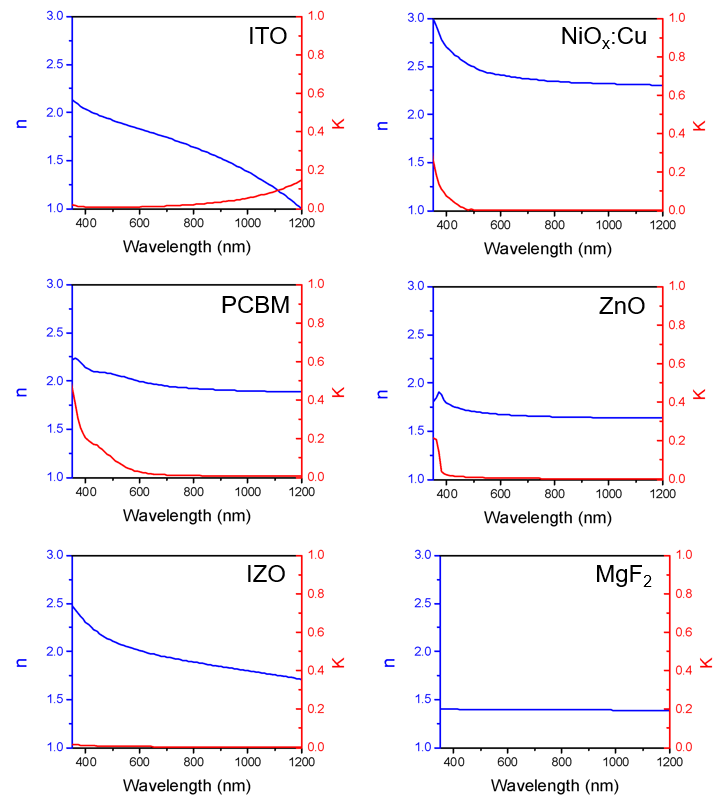
**

**Fig. S22.** Refractive indices of ITO, NiO*_x_*:Cu, PCBM, ZnO, IZO, and MgF_2_ thin films, determined by spectroscopic ellipsometry.

**Table S4.** The range of constituent layer thickness (*t*_sim_) investigated with optical simulations for the perovskite/Al-BSF Si tandem solar cell and summary of the equivalent current density (*j*_ph_) of each layer in the representative and optically optimized tandem cells.

| Layers | *t*_exp_ [nm] | *t*_sim_ [nm] | *j*_ph_ [mA cm^-2^] | |
| --- | --- | --- | --- | --- |
|  |  |  | Present | Optimized |
| Perovskite | 250 ± 9 | 250 | 16.3 | 18.5 |
| Si | 5.25×10^5^ | 5.25×10^5^ | 16.2 (19.8)^a^ | 18.5 (22.1)^a^ |
| Reflection | - | - | 5.0 | 3.0 |
| MgF_2_ | 105 ± 7 | 105 | 0.0 | 0.0 |
| IZO | 100 ± 5 | 10 – 150 (30)^b^ | 0.4 | 0.2 |
| ZnO-NP | 47 ± 2 | 10 | 0.4 | 0.1 |
| PCBM | 57 ± 8 | 10 | 2.3 | 0.4 |
| Cu:NiO*_x_* | 39 ± 6 | 10 – 80 | 0.0 | 0.0 |
| ITO | 20 ± 2 | 10 – 150 (10)^b^ | 0.5 | 0.4 |
| Al | 2.00×10^3^ | 2.00×10^3^ | 1.4 | 1.6 |

^a^The equivalent current density (*j*_ph_) without taking IQE drops into account. ^b^The IZO top and ITO interlayer thicknesses at which the surface reflection of the present tandem cell is minimized.

**Table S5.** PC1D parameters for the IQE simulations of the Al-BSF Si bottom cells.

| Parameters | Values |
| --- | --- |
|  |  |
| Bulk resistivity $(\Omega cm)$ | 3.0 |
| Bulk minority carrier lifetime (µs) | 435 |
| Front surface recombination velocity (FSRV) (cm/s) | 3.0$\times$10^5^ |
| Back surface recombination velocity (BSRV) (cm/s) | 10^2^ ~ 10^7^ |
| Interntal rear-sdie reflectance (%) | 85.0 |

To assess the optical and photovoltaic properties of the Si buttom cell and the BSRV values therefrom, single-junction Al-BSF Si solar cells were fabricated employing an ITO top electrode in a manner similar to the bottom cell in the tandem architecture. The front surface of Si wafers was polished before the fabrication process. The current-voltage characteristics of the device with the configuration of Al/Al-BSF/*p*-Si/*n^+^*^+^ Si/ITO/Ag-grid were measured under 1-sun-equivalent illumination as shown in **Fig. S23**a. The PV parameters determined therefrom were summarized in **Table S6**. **Fig. S23**b shows the EQE of the Si subcells with an ITO front layer measured using a custom-built equipment as well as the IQE of the Si bottom cell determined by normalizing the EQE curve to the total reflectance. Then, by fitting the EQE values simulated with PC1D to the measured one, we evaluted the BSRV and the front surface recombination velocity (FSRV) of the Al-BSF Si subcells, as shown in **Fig. S23**b. The IQE curve of the Si subcell was further simulated with PC1D and represented together with that determined experimentally. The equivalent current density of Si was thus adjusted taking the IQE into account. The adjusted *j*_ph_ of Si considering IQE depicted in **Fig. S23**b, 16.2 mA cm^–2^, was in good agreement with the measured *j*_SC,int_ (15.9 mA cm^–2^), indicating that the recombination loss at the back contact indeed dominates the performance of Al-BSF Si subcells.

**Fig. S23.** (a) Device structure and typical *j*–*V* characteristics under a standard AM 1.5G solar irradiation of planar (without texturing) Al-BSF Si solar cells employed as bottom cells in the tandem architecture. (b) Experimental (solid line) and simulated (dashed line) IQE and EQE spectra of the Si subcell along with the total reflectance (green line).

**Table S6**. Typical PV parameters of planar Al-BSF Si bottom cells with an ITO top electrode under simulated AM 1.5G illumination at 100 mW cm^–2^.

| *V*_OC_  [V] | *j*_SC_  [mA cm^−2^] | *FF*  [%] | *η*  [%] | *R*s  [Ohm cm^−2^] | Area  [cm^2^] |
| --- | --- | --- | --- | --- | --- |
| 0.57 ± 0.01 | 23.3 ± 0.1  (26.7 ± 0.3)^a^ | 76.2 ± 2.0 | 10.1 ± 0.2 | 0.89 ± 0.05 | 0.25 |

^a^ Integrated current density (j_SC,int_) obtained from EQE

The breakdown analysis of optical absorptances (**Fig. 8**a) further reveals that the reflection and parasitic absorption are significant causes of the photocurrent losses. The reflectance loss could be minimized by an elaborate optical design. In a wavelength region of *λ* < 800 nm, the front layers mostly affect the reflectance because incident light cannot penetrate the interfaces of the perovskite and Si subcells. On the other hand, in a wavelength range of *λ* > 800 nm, where the perovskite is transparent, the design of the interlayers between the perovskite and Si subcells is crucial. In a wavelength range of *λ* > 1000 nm, the backside of the Si bottom cell can affect the reflectance although its effect is limited in the current Al-BSF cell architecture. In this regard, we investigated the effect of the thicknesses of IZO top layer and ITO/Cu:NiO*_x_* interlayers on the reflectance to find the lowest reflectance conditions. In all simulations, the thicknesses of PCBM and ZnO-NPs layers were set to be 10 nm, while the thickness of the antireflection layer (MgF_2_) being fixed at 105 nm, as summarized in **Table S4**.

More specifically, in the wavelength range of *λ* < 800 nm, the reflectance is mainly influenced by the design of the front layers, *i.e.*, MgF_2_, IZO, ZnO, and PCBM. All of these layers beside MgF_2_ have similar *n* values having an average of 1.9 ± 0.2 at 550 nm. Therefore, the double layer antireflection design rule [98,99] can be applied with a combination of MgF_2_ based on the equations of $d_{1}=\lambda_{0}/4n_{1}$ and $d_{2}=\lambda_{0}/4n_{2}$. Here, $d_{1}$, $d_{2}$, $n_{1}$, $n_{2}$, and $\lambda_{0}$ denote the thickness of MgF_2_, the sum of the thicknesses of IZO, ZnO, and PCBM, the refractive index of MgF_2_, the average refractive index of IZO, ZnO, and PCBM, and the incident light wavelength. At *e.g.*, 550 nm, with the $n_{1}$ and $n_{2}$ values of 1.4 and 1.9, the $d_{1}$ and $d_{2}$ are calculated as 98 and 72 nm, respectively. According to this rough estimation, the lowest reflectance is expected at the IZO thickness of ~52 nm. Our simulation shows, however, that the lowest reflectance is found at 30-nm-thick IZO as shown in **Fig. 24**a, which is slightly thinner than estimated. On the other hand, since the ITO interlayer lying between the perovskite and Si layers has lower *n* values compared to the perovskite and Si, the reflection at the perovskite/Cu:NiO*_x_*/ITO and ITO/Si interfaces increases as the ITO thickness increases until reaching the quarter-wave optical thickness (= $\lambda_{0}$/4*n*). The simulation results in **Fig. S24** show that, in the thickness range of ITO examined, a thinner ITO interlayer can achieve lower reflection. In contrast, Cu:NiO*_x_* has a higher *n* value of ~2.4 at 550 nm, which is similar to that of perovskite. This tolerable *n* mismatch between Cu:NiO*_x_* and perovskite results in a negligible Cu:NiO*_x_* thickness dependence of the reflectance as illustrated in **Fig. S24**b. In sum, as can be seen in **Fig. S24**a, the minimum reflectance can be achieved with a 30-nm-thick IZO top layer and a 10-nm-thick ITO interlayer.

**Fig. S24.** Simulated reflectance loss contour map of the present perovskite/Al-BSF Si tandem solar cells **(**a) as a function of the thicknesses of IZO top TCO and ITO recombination layers with the Cu:NiO*_x_* layer thickness being fixed at 10 nm and (b) as a function of the thicknesses of ITO and Cu:NiO*_x_* layers with the thickness of IZO being fixed at 30 nm.

In **Fig. S25**, to visualize the effect of ITO interlayer thickness on the reflectance of the tandem cells, the normalized light intensity ($E^{2}/E_{0}^{2}$) profiles inside perovskite subcells were simulated at different wavelengths of light using a transfer matrix algorithm for various interlayer ITO thicknesses. For the sake of simplicity, the Si layer was assumed to be semi-infinite. At short wavelengths from 400 to 500 nm, the normalized light intensity profile barely changes by varying the ITO thickness. This is because short wavelength light cannot reach the rear side of the perovskite layer due to the limited optical penetration depth. In contrast, long wavelength light of, *e.g.*, 800 nm, can transmit to and is reflected from the rear side to interfere with the incident light, resulting in the large amplitude of the normalized light intensity [100,101]. One can see in **Fig. S25**c that the amplitude of the *E*-field significantly increases with increasing the ITO interlayer thickness. Namely, to reduce the reflectance loss at wavelength above 700 nm, the ITO interlayer thickness should be reduced, which can be seen more clearly in the simulated total reflectance spectra of the tandem cell in **Fig. S25**d.

**Fig. S25.** Normalized light intensity distributions along the vertical direction of the perovskite/Al-BSF Si tandem structure simulated for various ITO recombination layer thicknesses at light wavelengths of (a) 400 nm, (b) 500 nm, and (c) 800 nm, respectively. (d) Simulated total reflectance of the tandem structure for various ITO thicknesses.

**References**

[1] G. Haacke, New figure of merit for transparent conductors. J. Appl. Phys. **47**, 4086–4089 (1976). https://doi.org/10.1063/1.323240

[2] L. Qiao, T. Ye, T. Wang, W. Kong, R. Sun, L. Zhang, P. Wang, Z. Ge, Y. Peng, X. Zhang, M. Xu, X. Yan, J. Yang, X. Zhang, F. Zeng, L. Han, X. Yang, Freezing halide segregation under intense light for photostable perovskite/silicon tandem solar cells. Adv. Energy Mater. **14**, 2302983 (2024). https://doi.org/10.1002/aenm.202302983

[3] X. Luo, H. Luo, H. Li, R. Xia, X. Zheng, Z. Huang, Z. Liu, H. Gao, X. Zhang, S. Li, Z. Feng, Y. Chen, H. Tan, Efficient perovskite/silicon tandem solar cells on industrially compatible textured silicon. Adv. Mater. **35**, 2207883 (2023). https://doi.org/10.1002/adma.202207883

[4] R.L.Z. Hoye, K.A. Bush, F. Oviedo, S.E. Sofia, M. Thway, X. Li, Z. Liu, J. Jean, J.P. Mailoa, A. Osherov, F. Lin, A.F. Palmstrom, V. Bulović, M.D. McGehee, I.M. Peters, T. Buonassisi, Developing a robust recombination contact to realize monolithic perovskite tandems with industrially common p-type silicon solar cells. IEEE J. Photovolt. **8**, 1023–1028 (2018). https://doi.org/10.1109/JPHOTOV.2018.2820509

[5] K.A. Bush, A.F. Palmstrom, Z.J. Yu, M. Boccard, R. Cheacharoen, J.P. Mailoa, D.P. McMeekin, R.L.Z. Hoye, C.D. Bailie, T. Leijtens, I.M. Peters, M.C. Minichetti, N. Rolston, R. Prasanna, S. Sofia, D. Harwood, W. Ma, F. Moghadam, H.J. Snaith, T. Buonassisi, Z.C. Holman, S.F. Bent, M.D. McGehee, 23.6%-efficient monolithic perovskite/silicon tandem solar cells with improved stability. Nat. Energy **2**, 17009 (2017). https://doi.org/10.1038/NENERGY.2017.9

[6] M. Najafi, V. Zardetto, D. Zhang, D. Koushik, M.S. Dörenkämper, M. Creatore, R. Andriessen, P. Poodt, S. Veenstra, Highly efficient and stable semi-transparent p-i-n planar perovskite solar cells by atmospheric pressure spatial atomic layer deposited ZnO. Solar RRL **2**, 1800147 (2018). https://doi.org/10.1002/solr.201800147

[7] I.J. Park, J.H. Park, S.G. Ji, M.-A. Park, J.H. Jang, J.Y. Kim, A three-terminal monolithic perovskite/si tandem solar cell characterization platform. Joule **3**, 807–818 (2019). https://doi.org/10.1016/j.joule.2018.11.017

[8] H. Liang, J. Feng, C.D. Rodríguez-Gallegos, M. Krause, X. Wang, E. Alvianto, R. Guo, H. Liu, R.K. Kothandaraman, R. Carron, A.N. Tiwari, I.M. Peters, F. Fu, Y. Hou, 29.9%-efficient, commercially viable perovskite/CuInSe_2_ thin-film tandem solar cells. Joule **7**, 2859–2872 (2023). https://doi.org/10.1016/j.joule.2023.10.007

[9] S. Yoon, H.U. Ha, H.-J. Seok, H.-K. Kim, D.-W. Kang, Highly efficient and reliable semitransparent perovskite solar cells via top electrode engineering. Adv. Funct. Mater. **32**, 2111760 (2022). https://doi.org/10.1002/adfm.202111760

[10] D. Zhang, M. Najafi, V. Zardetto, M. Dörenkämper, X. Zhou, S. Veenstra, L.J. Geerligs, T. Aernouts, R. Andriessen, High efficiency 4-terminal perovskite/c-Si tandem cells. Sol. Energy Mater. Sol. Cells **188**, 1–5 (2018). https://doi.org/10.1016/j.solmat.2018.07.032

[11] Z. Ying, Y. Zhu, X. Feng, J. Xiu, R. Zhang, X. Ma, Y. Deng, H. Pan, Z. He, Sputtered indium-zinc oxide for buffer layer free semitransparent perovskite photovoltaic devices in perovskite/silicon 4T-tandem solar cells. Adv. Mater. Interfaces **8**, 2001604 (2021). https://doi.org/10.1002/admi.202001604

[12] S.-H. Lim, H.-J. Seok, M.-J. Kwak, D.-H. Choi, S.-K. Kim, D.-H. Kim, H.-K. Kim, Semi-transparent perovskite solar cells with bidirectional transparent electrodes. Nano Energy **82**, 105703 (2021). https://doi.org/10.1016/j.nanoen.2020.105703

[13] G.E. Eperon, T. Leijtens, K.A. Bush, R. Prasanna, T. Green, J.T.-W. Wang, D.P. McMeekin, G. Volonakis, R.L. Milot, R. May, A. Palmstrom, D.J. Slotcavage, R.A. Belisle, J.B. Patel, E.S. Parrott, R.J. Sutton, W. Ma, F. Moghadam, B. Conings, A. Babayigit, H.-G. Boyen, S. Bent, F. Giustino, L.M. Herz, M.B. Johnston, M.D. McGehee, H.J. Snaith, Perovskite-perovskite tandem photovoltaics with optimized band gaps. Science **354**, 861–865 (2016). https://doi.org/10.1126/science.aaf9717

[14] S.-H. Lim, H.-J. Seok, D.-H. Choi, S.-K. Kim, D.-H. Kim, H.-K. Kim, Room temperature processed transparent amorphous InGaTiO cathodes for semi-transparent perovskite solar cells. ACS Appl. Mater. Interfaces **13**, 27353–27363 (2021). https://doi.org/10.1021/acsami.1c02327

[15] J. Roger, L.K. Schorn, M. Heydarian, A. Farag, T. Feeney, D. Baumann, H. Hu, F. Laufer, W. Duan, K. Ding, A. Lambertz, P. Fassl, M. Worgull, U.W. Paetzold, Laminated monolithic perovskite/silicon tandem photovoltaics. Adv. Energy Mater. **12**, 2200961 (2022). https://doi.org/10.1002/aenm.202200961

[16] Q. Wen, C. Duan, F. Zou, D. Luo, J. Li, Z. Liu, J. Wang, K. Yan, All-inorganic CsPb_1-x_Sn_x_I_2_Br perovskites mediated by dicyandiamide additive for efficient 4-terminal tandem solar cell. Chem. Eng. J. **452**, 139697 (2023). https://doi.org/10.1016/j.cej.2022.139697

[17] Z. Ying, W. Chen, Y. Lin, Z. He, T. Chen, Y. Zhu, X. Zhang, X. Yang, A.B. Djurišić, Z. He, Supersmooth Ta_2_O_5_/Ag/polyetherimide film as the rear transparent electrode for high performance semitransparent perovskite solar cells. Adv. Optical Mater. **7**, 1801409 (2019). https://doi.org/10.1002/adom.201801409

[18] E. Aydin, M. De Bastiani, X. Yang, M. Sajjad, F. Aljamaan, Y. Smirnov, M.N. Hedhili, W. Liu, T.G. Allen, L. Xu, E. Van Kerschaver, M. Morales-Masis, U. Schwingenschlögl, S. De Wolf, Zr-doped indium oxide (IZRO) transparent electrodes for perovskite-based tandem solar cells. Adv. Funct. Mater. **29**, 1901741 (2019). https://doi.org/10.1002/adfm.201901741

[19] K. Wang, M. Neophytou, E. Aydin, M. Wang, T. Laurent, G.T. Harrison, J. Liu, W. Liu, M. De Bastiani, J.I. Khan, T.D. Anthopoulos, F. Laquai, S. De Wolf, Triarylphosphine oxide as cathode interfacial material for inverted perovskite solar cells. Adv. Mater. Interfaces **6**, 1900434 (2019). https://doi.org/10.1002/admi.201900434

[20] Q. Xue, Y. Bai, M. Liu, R. Xia, Z. Hu, Z. Chen, X.-F. Jiang, F. Huang, S. Yang, Y. Matsuo, H.-L. Yip, Y. Cao, Dual interfacial modifications enable high performance semitransparent perovskite solar cells with large open circuit voltage and fill factor. Adv. Energy Mater. **7**, 1602333 (2017). https://doi.org/10.1002/aenm.201602333

[21] M.B. Islam, M. Yanagida, Y. Shirai, Y. Nabetani, K. Miyano, Highly stable semi-transparent MAPbI_3_ perovskite solar cells with operational output for 4000 h. Sol. Energy Mater. Sol. Cells **195**, 323–329 (2019). https://doi.org/10.1016/j.solmat.2019.03.004

[22] J. Liu, Y. He, L. Ding, H. Zhang, Q. Li, L. Jia, J. Yu, T.W. Lau, M. Li, Y. Qin, X. Gu, F. Zhang, Q. Li, Y. Yang, S. Zhao, X. Wu, J. Liu, T. Liu, Y. Gao, Y. Wang, X. Dong, H. Chen, P. Li, T. Zhou, M. Yang, X. Ru, F. Peng, S. Yin, M. Qu, D. Zhao, Z. Zhao, M. Li, P. Guo, H. Yan, C. Xiao, P. Xiao, J. Yin, X. Zhang, Z. Li, B. He, X. Xu, Perovskite/silicon tandem solar cells with bilayer interface passivation. Nature **635**, 596–603 (2024). https://doi.org/10.1038/s41586-024-07997-7

[23] E. Ugur, A.A. Said, P. Dally, S. Zhang, C.E. Petoukhoff, D. Rosas-Villalva, S. Zhumagali, B.K. Yildirim, A. Razzaq, S. Sarwade, A. Yazmaciyan, D. Baran, F. Laquai, C. Deger, I. Yavuz, T.G. Allen, E. Aydin, S. De Wolf, Enhanced cation interaction in perovskites for efficient tandem solar cells with silicon. Science **385**, 533–538 (2024). https://doi.org/10.1126/science.adp1621

[24] E. Aydin, E. Ugur, B.K. Yildirim, T.G. Allen, P. Dally, A. Razzaq, F. Cao, L. Xu, B. Vishal, A. Yazmaciyan, A.A. Said, S. Zhumagali, R. Azmi, M. Babics, A. Fell, C. Xiao, S. De Wolf, Enhanced optoelectronic coupling for perovskite/silicon tandem solar cells. Nature **623**, 732–738 (2023). https://doi.org/10.1038/s41586-023-06667-4

[25] S. Mariotti, E. Köhnen, F. Scheler, K. Sveinbjörnsson, L. Zimmermann, M. Piot, F. Yang, B. Li, J. Warby, A. Musiienko, D. Menzel, F. Lang, S. Keßler, I. Levine, D. Mantione, A. Al-Ashouri, M.S. Härtel, K. Xu, A. Cruz, J. Kurpiers, P. Wagner, H. Köbler, J. Li, A. Magomedov, D. Mecerreyes, E. Unger, A. Abate, M. Stolterfoht, B. Stannowski, R. Schlatmann, L. Korte, S. Albrecht, Interface engineering for high-performance, triple-halide perovskite–silicon tandem solar cells. Science **381**, 63–69 (2023). https://doi.org/10.1126/science.adf5872

[26] Z. Liu, Z. Xiong, S. Yang, K. Fan, L. Jiang, Y. Mao, C. Qin, S. Li, L. Qiu, J. Zhang, F.R. Lin, L. Fei, Y. Hua, J. Yao, C. Yu, J. Zhou, Y. Chen, H. Zhang, H. Huang, A.K.-Y. Jen, K. Yao, Strained heterojunction enables high-performance, fully textured perovskite/silicon tandem solar cells. Joule **8**, 2834–2850 (2024). https://doi.org/10.1016/j.joule.2024.06.015

[27] X.Y. Chin, D. Turkay, J.A. Steele, S. Tabean, S. Eswara, M. Mensi, P. Fiala, C.M. Wolff, A. Paracchino, K. Artuk, D. Jacobs, Q. Guesnay, F. Sahli, G. Andreatta, M. Boccard, Q. Jeangros, C. Ballif, Interface passivation for 31.25%-efficient perovskite/silicon tandem solar cells. Science **381**, 59–63 (2023). https://doi.org/10.1126/science.adg0091

[28] F. Pei, Y. Chen, Q. Wang, L. Li, Y. Ma, H. Liu, Y. Duan, T. Song, H. Xie, G. Liu, N. Yang, Y. Zhang, W. Zhou, J. Kang, X. Niu, K. Li, F. Wang, M. Xiao, G. Yuan, Y. Wu, C. Zhu, X. Wang, H. Zhou, Y. Wu, Q. Chen, A binary 2D perovskite passivation for efficient and stable perovskite/silicon tandem solar cells. Nat. Commun. **15**, 7024 (2024). https://doi.org/10.1038/s41467-024-51345-2

[29] Z. Zhu, S. Yuan, K. Mao, H. Meng, F. Cai, T. Li, X. Feng, H. Guo, L. Tang, J. Xu, Low-temperature atomic layer deposition of hole transport layers for enhanced performance and scalability in textured perovskite/silicon tandem solar cells. Adv. Energy Mater. **14**, 2402365 (2024). https://doi.org/10.1002/aenm.202402365

[30] J. Liu, B. Shi, Q. Xu, Y. Li, Y. Li, P. Liu, Z. SunLi, X. Wang, C. Sun, W. Han, D. Li, S Wang, D. Zhang, G. Li, X. Du, Y. Zhao, X. Zhang, Textured perovskite/silicon tandem solar cells achieving over 30% efficiency promoted by 4-fluorobenzylamine hydroiodide. Nano-Micro Lett. **16**, 189 (2024). https://doi.org/10.1007/s40820-024-01406-4

[31] B. Li, M. Härtel, A. Al-Ashouri, M. Simmonds, I. Taupitz, L. Kegelmann, E. Jarzembowski, F. Frühauf, E. Köhnen, L. Korte, F. Fertig, J. Müller, S. Albrecht. Atomic-layer-deposition-free monolithic perovskite/silicon tandem solar cell reaching 29.91% power conversion on industrial PERX/TOPCon-like silicon bottom cells. ACS Energy Lett. **9**, 4550–4556 (2024). https://doi.org/10.1021/acsenergylett.4c01502

[32] P. Tockhorn, J. Sutter, A. Cruz, P. Wagner, K. Jäger, D. Yoo, F. Lang, M. Grischek, B. Li, A. Al-Ashouri, E. Köhnen, M. Stolterfoht, D. Neher, R. Schlatmann, B. Rech, B. Stannowski, S. Albrecht, C. Becker, Nano-optical designs for high-efficiency monolithic perovskite–silicon tandem solar cells. Nat. Nanotechnol. **17**, 1214–1221 (2002). https://doi.org/10.1038/s41565-022-01228-8

[33] S.G. Ji, I.J. Park, H. Chang, J.H. Park, G.P. Hong, B.K. Choi, J.H. Jang, Y.J. Choi, H.W. Lim, Y.J. Ahn, S.J. Park, K.T. Nam, T. Hyeon, J. Park, D.H. Kim, J.Y. Kim, Stable pure-iodide wide-band-gap perovskites for efficient Si tandem cells via kinetically controlled phase evolution. Joule **6**, 2390–2402 (2022). https://doi.org/10.1016/j.joule.2022.08.006

[34] J. Liu, M. De Bastiani, E. Aydin, G.T. Harrison, Y. Gao, R.R. Pradhan, M.K. Eswaran, M. Mandal, W. Yan, A. Seitkhan, M. Babics, A.S. Subbiah, E. Ugur, F. Xu, L. Xu, M. Wang, A. Ur Rehman, A. Razzaq, J. Kang, R. Azmi, A.A. Said, F.H. Isikgor, T.G. Allen, D. Andrienko, U. Schwingenschlögl, F. Laquai, S. De Wolf, Efficient and stable perovskite-silicon tandem solar cells through contact displacement by MgF*_x_*. Science **377**, 302–306 (2022). https://doi.org/10.1126/science.abn8910

[35] K. Yamamoto, R. Mishima, H. Uzu, D. Adachi, High efficiency perovskite/heterojunction crystalline silicon tandem solar cells: Towards industrial-sized cell and module. Jpn. J. Appl. Phys. **62**, SK1021 (2023). https://doi.org/10.35848/1347-4065/acc593

[36] A. Al-Ashouri, E. Köhnen, B. Li, A. Magomedov, H. Hempel, P. Caprioglio, J.A. Márquez, A.B.M. Vilches, E. Kasparavicius, J.A. Smith, N. Phung, D. Menzel, M. Grischek, L. Kegelmann, D. Skroblin, C. Gollwitzer, T. Malinauskas, M. Jošt, G. Matič, B. Rech, R. Schlatmann, M. Topič, L. Korte, A. Abate, B. Stannowski, D. Neher, M. Stolterfoht, T. Unold, V. Getautis, S. Albrecht, Monolithic perovskite/silicon tandem solar cell with >29% efficiency by enhanced hole extraction. Science **370**, 1300–1309 (2020). https://doi.org/10.1126/science.abd4016

[37] J. Li, B. Farhadi, S. Liu, L. Liu, H. Wang, M. Du, L. Yang, S. Bao, S.-T. Zhang, D. Li, K. Wang, S. Liu, Built-in field manipulation through a perovskite homojunction for efficient monolithic perovskite/silicon tandem solar cells. Nano Energy **129**, 109976 (2024). https://doi.org/10.1016/j.nanoen.2024.109976

[38] G. Wang, J. Zheng, W. Duan, J. Yang, M.A. Mahmud, Q. Lian, S. Tang, C. Liao, J. Bing, J. Yi, T.L. Leung, X. Cui, H. Chen, F. Jiang, Y. Huang, A. Lambertz, M. Jankovec, M. Topič, S. Bremner, Y.-Z. Zhang, C. Cheng, K. Ding, A. Ho-Baillie, Molecular engineering of hole-selective layer for high band gap perovskites for highly efficient and stable perovskite-silicon tandem solar cells. Joule **7**, 2583–2594 (2023). https://doi.org/10.1016/j.joule.2023.09.007

[39] L. Xu, J. Liu, F. Toniolo, M. De Bastiani, M. Babics, W. Yan, F. Xu, J. Kang, T. Allen, A. Razzaq, E. Aydin, S. De Wolf, Monolithic perovskite/silicon tandem photovoltaics with minimized cell-to-module losses by refractive-index engineering. ACS Energy Lett. **7**, 2370–2372 (2022). https://doi.org/10.1021/acsenergylett.2c01142

[40] J. Liu, E. Aydin, J. Yin, M. De Bastiani, F.H. Isikgor, A. ur Rehman, E. Yengel, E. Ugur, G.T. Harrison, M. Wang, Y. Gao, J.I. Khan, M. Babics, T.G. Allen, A.S. Subbiah, K. Zhu, X. Zheng, W. Yan, F. Xu, M. F. Salvador, O.M. Bakr, T.D. Anthopoulos, M. Lanza, O.F. Mohammed, F. Laquai, S. De Wolf, 28.2%-efficient, outdoor-stable perovskite/silicon tandem solar cell. Joule **5**, 3169–3186 (2021). https://doi.org/10.1016/j.joule.2021.11.003

[41] K. Sveinbjörnsson, B. Li, S. Mariotti, E. Jarzembowski, L. Kegelmann, A. Wirtz, F. Frühauf, A. Weihrauch, R. Niemann, L. Korte, F. Fertig, J.W. Müller, S. Albrecht, Monolithic perovskite/silicon tandem solar cell with 28.7% efficiency using industrial silicon bottom cells. ACS Energy Lett. **7**, 2654–2656 (2022). https://doi.org/10.1021/acsenergylett.2c01358

[42] H. Luo, X. Zheng, W. Kong, Z. Liu, H. Li, J. Wen, R. Xia, H. Sun, P. Wu, Y. Wang, Y. Mo, X. Luo, Z. Huang, J. Hong, Z. Chu, X. Zhang, G. Yang, Y. Chen, Z. Feng, J. Gao, H. Tan, Inorganic framework composition engineering for scalable fabrication of perovskite/silicon tandem solar cells. ACS Energy Lett. **8**, 4993–5002 (2023). https://doi.org/10.1021/acsenergylett.3c02002

[43] G. Yang, Z. Ni, Z.J. Yu, B.W. Larson, Z. Yu, B. Chen, A. Alasfour, X. Xiao, J.M. Luther, Z.C. Holman, J. Huang, Defect engineering in wide-bandgap perovskites for efficient perovskite–silicon tandem solar cells. Nat. Photonics **16**, 588–594 (2022). https://doi.org/10.1038/s41566-022-01033-8

[44] Q. Xu, B. Shi, Y. Li, L. Yan, W. Duan, Y. Li, R. Li, N. Ren, W. Han, J. Liu, Q. Huang, D. Zhang, H. Ren, S. Xu, C. Zhang, H. Zhuang, A. Lambertz, K. Ding, Y. Zhao, X. Zhang, Conductive passivator for efficient monolithic perovskite/silicon tandem solar cell on commercially textured silicon. Adv. Energy Mater. **12**, 2202404 (2022). https://doi.org/10.1002/aenm.202202404

[45] Z. Ying, Z. Yang, J. Zheng, H. Wei, L. Chen, C. Xiao, J. Sun, C. Shou, G. Qin, J. Sheng, Y. Zeng, B. Yan, X. Yang, J. Ye, Monolithic perovskite/black-silicon tandems based on tunnel oxide passivated contacts. Joule **6**, 2644–2661 (2022). https://doi.org/10.1016/j.joule.2022.09.006

[46] M. De Bastiani, R. Jalmood, J. Liu, C. Ossig, A. Vlk, K. Vegso, M. Babics, F.H. Isikgor, A.S. Selvin, R. Azmi, E. Ugur, S. Banerjee, A.J. Mirabelli, E. Aydin, T.G. Allen, A. Ur Rehman, E. Van Kerschaver, P. Siffalovic, M.E. Stuckelberger, M. Ledinsky, S. De Wolf, Monolithic perovskite/silicon tandems with >28% efficiency: Role of silicon-surface texture on perovskite properties. Adv. Funct. Mater. **33**, 2205557 (2023). https://doi.org/10.1002/adfm.202205557

[47] X. Zheng, J. Liu, T. Liu, E. Aydin, M. Chen, W. Yan, M. De Bastiani, T.G. Allen, S. Yuan, A.R. Kirmani, K.N. Baustert, M.F. Salvador, B. Turedi, A.Y. Alsalloum, K. Almasabi, K. Kotsovos, I. Gereige, L.-S. Liao, J.M. Luther, K.R. Graham, O.F. Mohammed, S. De Wolf, O.M. Bakr, ACS Energy Lett. **7**, 1987–1993 (2022). https://doi.org/10.1021/acsenergylett.2c00780

[48] B. Chen, P. Wang, R. Li, N. Ren, W. Han, Z. Zhu, J. Wang, S. Wang, B. Shi, J. Liu, P. Liu, Q. Huang, S. Xu, Y. Zhao, X. Zhang, Photoactivated p-doping of organic interlayer enables efficient perovskite/silicon tandem solar cells. ACS Energy Lett. **7**, 2771–2780 (2022). https://doi.org/10.1021/acsenergylett.2c01488

[49] Y. Wu, P. Zheng, J. Peng, M. Xu, Y. Chen, S. Surve, T. Lu, A.D. Bui, N. Li, W. Liang, L. Duan, B. Li, H. Shen, T. Duong, J. Yang, X. Zhang, Y. Liu, H. Jin, Q. Chen, T. White, K. Catchpole, H. Zhou, K. Weber, 27.6% perovskite/c-Si tandem solar cells using industrial fabricated TOPCon device. Adv. Energy Mater. **12**, 2200821 (2022). https://doi.org/10.1002/aenm.202200821

[50] Y.-Y. Xu, Y. Jiang, H.-Q. Du, X. Gao, Z.-Y. Qiang, C.-X. Wang, Z.-W. Tao, L.-H. Yang, R. Zhi, G.-J. Liang, H.-Y. Cai, M.U. Rothmann, Y.-B. Cheng, W. Li, Octahedral tilt enables efficient and stable fully vapor-deposited perovskite/silicon tandem cells. Adv. Funct. Mater. **34**, 2312037 (2024). https://doi.org/10.1002/adfm.202312037

[51] F.H. Isikgor, F. Furlan, J. Liu, E. Ugur, M.K. Eswaran, A.S. Subbiah, E. Yengel, M. De Bastiani, G.T. Harrison, S. Zhumagali, C.T. Howells, E. Aydin, M. Wang, N. Gasparini, T.G. Allen, A. ur Rehman, E. Van Kerschaver, D. Baran, I. McCulloch, T.D. Anthopoulos, U. Schwingenschlögl, F. Laquai, S. De Wolf, Concurrent cationic and anionic perovskite defect passivation enables 27.4% perovskite/silicon tandems with suppression of halide segregation. Joule **5**, 1566–1586 (2021). https://doi.org/10.1016/j.joule.2021.05.013

[52] J. Xu, C.C. Boyd, Z.J. Yu, A.F. Palmstrom, D.J. Witter, B.W. Larson, R.M. France, J. Werner, S.P. Harvey, E.J. Wolf, W. Weigand, S. Manzoor, M.F.A.M. van Hest, J.J. Berry, J.M. Luther, Z.C. Holman, M.D. McGehee, Triple-halide wide–band gap perovskites with suppressed phase segregation for efficient tandems. Science **367**, 1097–1104 (2020). http://doi.org/10.1126/science.aaz5074

[53] E. Aydin, J. Liu, E. Ugur, R. Azmi, G.T. Harrison, Y. Hou, B. Chen, S. Zhumagali, M. De Bastiani, M. Wang, W. Raja, T.G. Allen, A. ur Rehman, A.S. Subbiah, M. Babics, A. Babayigit, F.H. Isikgor, K. Wang, E. Van Kerschaver, L. Tsetseris, E.H. Sargent, F. Laquai, S. De Wolf, Ligand-bridged charge extraction and enhanced quantum efficiency enable efficient n–i–p perovskite/silicon tandem solar cells. Energy Environ. Sci. **14**, 4377–4390 (2021). https://doi.org/10.1039/D1EE01206A

[54] S. Zhang, J. Wang, N. Kalasariya, P. Dally, C. Deger, I. Yavuz, A. Razzaq, B. Vishal, A. Prasetio, D.S. Utomo, O. Karalis, H. Hempel, V. Hnapovskyi, Q. Liu, M. Babics, A.A. Said, A. Pininti, M. Stolterfoht, S. De Wolf, Mitigating buried-interface energy losses through multifunctional ligands in n–i–p perovskite/silicon tandem solar cells. ACS Energy Lett. **9**, 4633–4644 (2024). https://doi.org/10.1021/acsenergylett.4c01841

[55] D. Kim, H.J. Jung, I.J. Park, B.W. Larson, S.P. Dunfield, C. Xiao, J. Kim, J. Tong, P. Boonmongkolras, S.G. Ji, F. Zhang, S.R. Pae, M. Kim, S.B. Kang, V. Dravid, J.J. Berry, J.Y. Kim, K. Zhu, D.H. Kim, B. Shin, Efficient, stable silicon tandem cells enabled by anion-engineered wide-bandgap perovskites. Science **368**, 155–160 (2020). https://doi.org/10.1126/science.aba3433

[56] S. Zhumagali, F.H. Isikgor, P. Maity, J. Yin, E. Ugur, M. De Bastiani, A.S. Subbiah, A. J. Mirabelli, R. Azmi, G.T. Harrison, J. Troughton, E. Aydin, J. Liu, T. Allen, A. ur Rehman, D. Baran, O.F. Mohammed, S. De Wolf, Linked nickel oxide/perovskite interface passivation for high-performance textured monolithic tandem solar cells. Adv. Energy Mater. **11**, 2101662 (2021). https://doi.org/10.1002/aenm.202101662

[57] B. Chen, Z.J. Yu, S. Manzoor, S. Wang, W. Weigand, Z. Yu, G. Yang, Z. Ni, X. Dai, Z. C. Holman, J. Huang, Blade-coated perovskites on textured silicon for 26%-efficient monolithic perovskite/silicon tandem solar cells. Joule **4**, 850–864 (2020). https://doi.org/10.1016/j.joule.2020.01.008

[58] E. Köhnen, M. Jošt, A.B. Morales-Vilches, P. Tockhorn, A. Al-Ashouri, B. Macco, L. Kegelmann, L. Korte, B, Rech, R. Schlatmann, B. Stannowski, S. Albrecht, Highly efficient monolithic perovskite silicon tandem solar cells: Analyzing the influence of current mismatch on device performance. Sustain. Energy Fuels **3**, 1995–2005 (2019). https://doi.org/10.1039/C9SE00120D

[59] Y. Hou, E. Aydin, M. De Bastiani, C. Xiao, F.H. Isikgor, D.-J. Xue, B. Chen, H. Chen, B. Bahrami, A.H. Chowdhury, A. Johnston, S.-W. Baek, Z. Huang, M. Wei, Y. Dong, J. Troughton, R. Jalmood, A.J. Mirabelli, T.G. Allen, E. Van Kerschaver, M.I. Saidaminov, D. Baran, Q. Qiao, K. Zhu, S. De Wolf, E.H. Sargent, Efficient tandem solar cells with solution-processed perovskite on textured crystalline silicon. Science **367**, 1135–1140 (2020). https://doi.org/10.1126/science.aaz3691

[60] F. Sahli, J. Werner, B.A. Kamino, M. Bräuninger, R. Monnard, B. Paviet-Salomon, L. Barraud, L. Ding, J.J.D. Leon, D. Sacchetto, G. Cattaneo, M. Despeisse, M. Boccard, S. Nicolay, Q. Jeangros, B. Niesen, C. Ballif, Fully textured monolithic perovskite/silicon tandem solar cells with 25.2% power conversion efficiency. Nat. Mater. **17**, 820–826 (2018). https://doi.org/10.1038/s41563-018-0115-4

[61] M. Jošt, E. Köhnen, A.B. Morales-Vilches, B. Lipovšek, K. Jäger, B. Macco, A. Al-Ashouri, J. Krč, L. Korte, B. Rech, R. Schlatmann, M. Topič, B. Stannowski, S. Albrecht, Textured interfaces in monolithic perovskite/silicon tandem solar cells: Advanced light management for improved efficiency and energy yield. Energy Environ. Sci. **11**, 3511–3523 (2018). https://doi.org/10.1039/C8EE02469C

[62] B. Chen, P. Wang, R. Li, N. Ren, Y. Chen, W. Han, L. Yan, Q. Huang, D. Zhang, Y. Zhao, X. Zhang, Composite electron transport layer for efficient N-I-P type monolithic perovskite/silicon tandem solar cells with high open-circuit voltage. J. Energy Chem. **63**, 461–467 (2021). https://doi.org/10.1016/j.jechem.2021.07.018

[63] L. Mazzarella, Y.-H. Lin, S. Kirner, A.B. Morales-Vilches, L. Korte, S. Albrecht, E. Crossland, B. Stannowski, C. Case, H.J. Snaith, R. Schlatmann, Infrared light management using a nanocrystalline silicon oxide interlayer in monolithic perovskite/silicon heterojunction tandem solar cells with efficiency above 25%. Adv. Energy Mater. **9**, 1803241 (2019). https://doi.org/10.1002/aenm.201803241

[64] G. Nogay, F. Sahli, J. Werner, R. Monnard, M. Boccard, M. Despeisse, F-J. Haug, Q. Jeangros, A. Ingenito, C. Ballif, 25.1%-efficient monolithic perovskite/silicon tandem solar cell based on a *p*-type monocrystalline textured silicon wafer and high-temperature passivating contacts. ACS Energy Lett. **4**, 844–845 (2019). https://doi.org/10.1021/acsenergylett.9b00377

[65] B. Chen, Z. Yu, K. Liu, X. Zheng, Y. Liu, J. Shi, D. Spronk, P.N. Rudd, Z. Holman, J. Huang, Grain engineering for perovskite/silicon monolithic tandem solar cells with efficiency of 25.4%. Joule **3**, 177–190 (2019). https://doi.org/10.1016/j.joule.2018.10.003

[66] M. De Bastiani, A.J. Mirabelli, Y. Hou, F. Gota, E. Aydin, T.G. Allen, J. Troughton, A. S. Subbiah, F.H. Isikgor, J. Liu, L. Xu, B. Chen, E. Van Kerschaver, D. Baran, B. Fraboni, M.F. Salvador, U.W. Paetzold, E.H. Sargent, S. De Wolf, Efficient bifacial monolithic perovskite/silicon tandem solar cells via bandgap engineering. Nat. Energy **6**, 167–175 (2021). https://doi.org/10.1038/s41560-020-00756-8

[67] E. Aydin, T.G. Allen, M. De Bastiani, L. Xu, J. Ávila, M. Salvador, E. Van Kerschaver, S. De Wolf, Interplay between temperature and bandgap energies on the outdoor performance of perovskite/silicon tandem solar cells. Nat. Energy **5**, 851–859 (2020). https://doi.org/10.1038/s41560-020-00687-4

[68] P.S.C. Schulze, A.J. Bett, M. Bivour, P. Caprioglio, F.M. Gerspacher, Ö.Ş. Kabaklı, A. Richter, M. Stolterfoht, Q. Zhang, D. Neher, M. Hermle, H. Hillebrecht, S.W. Glunz, J.C. Goldschmidt, 25.1% high-efficiency monolithic perovskite silicon tandem solar cell with a high bandgap perovskite absorber. Solar RRL **4**, 2000152 (2020). https://doi.org/10.1002/solr.202000152

[69] K.A. Bush, S. Manzoor, K. Frohna, Z.J. Yu, J.A. Raiford, A.F. Palmstrom, H.-P. Wang, R. Prasanna, S.F. Bent, Z.C. Holman, M.D. McGehee, Minimizing current and voltage losses to reach 25% efficient monolithic two-terminal perovskite–silicon tandem solar cells. ACS Energy Lett. **3**, 2173–2180 (2018). https://doi.org/10.1021/acsenergylett.8b01201

[70] H. Shen, S.T. Omelchenko, D.A. Jacobs, S. Yalamanchili, Y. Wan, D. Yan1, P. Phang, T. Duong, Y. Wu, Y. Yin, C. Samundsett, J. Peng, N. Wu, T.P. White, G.G. Andersson, N.S. Lewis, K.R. Catchpole, In situ recombination junction between p-Si and TiO_2_ enables high-efficiency monolithic perovskite/Si tandem cells. Sci. Adv. **4**, eaau9711 (2018). https://doi.org/10.1126/sciadv.aau9711

[71] M. Roß, S. Severin, M.B. Stutz, P. Wagner, H. Köbler, M. Favin-Lévêque, A. Al-Ashouri, P. Korb, P. Tockhorn, A. Abate, B. Stannowski, B. Rech, S. Albrecht, Co-evaporated formamidinium lead iodide based perovskites with 1000 h constant stability for fully textured monolithic perovskite/silicon tandem solar cells. Adv. Energy Mater. **11**, 2101460 (2021). https://doi.org/10.1002/aenm.202101460

[72] S. Lee, C.U. Kim, S. Bae, Y. Liu, Y.I. Noh, Z. Zhou, P.W. Leu, K.J. Choi, J.-K. Lee, Improving light absorption in a perovskite/Si tandem solar cell via light scattering and UV-down shifting by a mixture of SiO_2_ nanoparticles and phosphors. Adv. Funct. Mater. **32**, 2204328 (2022). https://doi.org/10.1002/adfm.202204328

[73] J. Zheng, H. Mehrvarz, C. Liao, J. Bing, X. Cui, Y. Li, V.R. Gonçales, C.F.J. Lau, D.S. Lee, Y. Li, M. Zhang, J. Kim, Y. Cho, L.G. Caro, S. Tang, C. Chen, S. Huang, A.W.Y. Ho-Baillie, Large-area 23%-efficient monolithic perovskite/homojunction-silicon tandem solar cell with enhanced UV stability using down-shifting material. ACS Energy Lett. **4**, 2623–2631 (2019). https://doi.org/10.1021/acsenergylett.9b01783

[74] S. Wang, Wang, B. Chen, R. Li, N. Ren, Y. Li, B. Shi, Q. Huang, Y. Zhao, M. Grätzel, X. Zhang, Suppressed recombination for monolithic inorganic perovskite/silicon tandem solar cells with an approximate efficiency of 23%. eScience **2**, 339–346 (2022). https://doi.org/10.1016/j.esci.2022.04.001

[75] S. Zhu, F. Hou, W. Huang, X. Yao, B. Shi, Q. Ren, J. Chen, L. Yan, S. An, Z. Zhou, H. Ren, C. Wei, Q. Huang, Y. Li, G. Hou, X. Chen, Y. Ding, G. Wang, B. Li, Y. Zhao, X. Zhang, Solvent engineering to balance light absorbance and transmittance in perovskite for tandem solar cells. Solar RRL **2**, 1800176 (2018). https://doi.org/10.1002/solr.201800176

[76] F. Sahli, B.A. Kamino, J. Werner, M. Bräuninger, B. Paviet-Salomon, L. Barraud, R. Monnard, J.P. Seif, A. Tomasi, Q. Jeangros, A. Hessler-Wyser, S. De Wolf, M. Despeisse, S. Nicolay, B. Niesen, C. Ballif, Improved optics in monolithic perovskite/silicon tandem solar cells with a nanocrystalline silicon recombination junction. Adv. Energy Mater. **8**, 1701609 (2017). https://doi.org/10.1002/aenm.201701609

[77] Y. Wu, D. Yan, J. Peng, T. Duong, Y. Wan, S.P. Phang, H. Shen, N. Wu, C. Barugkin, X. Fu, S. Surve, D. Grant D. Walter, T.P. White, K.R. Catchpole, K.J. Weber, Monolithic perovskite/silicon-homojunction tandem solar cell with over 22% efficiency. Energy Environ. Sci. **10**, 2472–2479 (2017). https://doi.org/10.1039/C7EE02288C

[78] Z. Qiu, Z. Xu, N. Li, N. Zhou, Y. Chen, X. Wan, J. Liu, N. Li, X. Hao, P. Bi, Q. Chen, B. Cao, H. Zhou, Monolithic perovskite/Si tandem solar cells exceeding 22% efficiency via optimizing top cell absorber. Nano Energy **53**, 798–807 (2018). https://doi.org/10.1016/j.nanoen.2018.09.052

[79] J. Zheng, H. Mehrvarz, F.-J. Ma, C.F.J. Lau, M.A. Green, S. Huang, A.W.Y. Ho-Baillie, 21.8% efficient monolithic perovskite/homo-junction-silicon tandem solar cell on 16 cm^2^. ACS Energy Lett. **3**, 2299–2300 (2018). https://doi.org/10.1021/acsenergylett.8b01382

[80] A.J. Bett, P.S.C. Schulze, K.M. Winkler, Ö.S. Kabakli, I. Ketterer, L.E. Mundt, S.K. Reichmuth, G. Siefer, L. Cojocaru, L. Tutsch, M. Bivour, M. Hermle, S.W. Glunz, J.C. Goldschmidt, Two-terminal perovskite silicon tandem solar cells with a high-bandgap perovskite absorber enabling voltages over 1.8 V. Prog. Photovolt. Res. Appl. **28**, 99–110 (2019). https://doi.org/10.1002/pip.3208

[81] C. McDonald, H. Sai, V. Svrcek, A. Kogo, T. Miyadera, T.N. Murakami, M. Chikamatsu, Y. Yoshida, T. Matsui, *In situ* grown nanocrystalline Si recombination junction layers for efficient perovskite–Si monolithic tandem solar cells: Toward a simpler multijunction architecture. ACS Appl. Mater. Interfaces **14**, 33505–33514 (2022). https://doi.org/10.1021/acsami.2c05662

[82] J. Werner, C.-H. Weng, A. Walter, L. Fesquet, J.P. Seif, S. De Wolf, B. Niesen, C. Ballif, Efficient monolithic perovskite/silicon tandem solar cell with cell area >1 cm^2^. J. Phys. Chem. Lett. **7**, 161–166 (2016). https://doi.org/10.1021/acs.jpclett.5b02686

[83] S. Mariotti, K. Jäger, M. Diederich, M.S. Härtel, B. Li, K. Sveinbjörnsson, S. Kajari-Schröder, R. Peibst, S. Albrecht, L. Korte, T. Wietler, Monolithic perovskite/silicon tandem solar cells fabricated using industrial p-type polycrystalline silicon on oxide/passivated emitter and rear cell silicon bottom cell technology. Solar RRL **6**, 2101066 (2022). https://doi.org/10.1002/solr.202101066

[84] C.U. Kim, J.C. Yu, E.D. Jung, I.Y. Choi, W. Park, H. Lee, I. Kim, D.-K. Lee, K.K. Hong, M.H. Song, K.J. Choi, Optimization of device design for low cost and high efficiency planar monolithic perovskite/silicon tandem solar cells. Nano Energy **60**, 213–221 (2019). https://doi.org/10.1016/j.nanoen.2019.03.056

[85] J. Zheng, C.F.J. Lau, H. Mehrvarz, F.-J. Ma, Y. Jiang, X. Deng, A. Soeriyadi, J. Kim, M. Zhang, L. Hu, X. Cui, D.S. Lee, J. Bing, Y. Cho, C. Chen, M.A. Green, S. Huang, A.W.Y. Ho-Baillie, Large area efficient interface layer free monolithic perovskite/homo-junction-silicon tandem solar cell with over 20% efficiency. Energy Environ. Sci. **11**, 2432–2443 (2018). https://doi.org/10.1039/C8EE00689J

[86] R. Fan, N. Zhou, L. Zhang, R. Yang, Y. Meng, L. Li, T. Guo, Y. Chen, Z. Xu, G. Zheng, Y. Huang, L. Li, L. Qin, X. Qiu, Q. Chen, H. Zhou, Toward full solution processed perovskite/Si monolithic tandem solar device with PCE exceeding 20%. Solar RRL **1**, 1700149 (2017). https://doi.org/10.1002/solr.201700149

[87] J. Werner, L. Barraud, A. Walter, M. Bräuninger, F. Sahli, D. Sacchetto, N. Tétreault, B. Paviet-Salomon, S.-J. Moon, C. Allebé, M. Despeisse, S. Nicolay, S. De Wolf, B. Niesen, C. Ballif, Efficient near-infrared-transparent perovskite solar cells enabling direct comparison of 4-terminal and monolithic perovskite/silicon tandem cells. ACS Energy Lett. **1**, 474–480 (2016). https://doi.org/10.1021/acsenergylett.6b00254

[88] S. An, P. Chen, F. Hou, Q. Wang, H. Pan, X. Chen, X. Lu, Y. Zhao, Q. Huang, X. Zhang, Cerium-doped indium oxide transparent electrode for semi-transparent perovskite and perovskite/silicon tandem solar cells. Sol. Energy **196**, 409–418 (2020). https://doi.org/10.1016/j.solener.2019.12.040

[89] S. Albrecht, M. Saliba, J.P.C. Baena, F. Lang, L. Kegelmann, M. Mews, L. Steier, A. Abate, J. Rappich, L. Korte, R. Schlatmann, M.K. Nazeeruddin, A. Hagfeldt, M. Grätzel, B. Rech, Monolithic perovskite/silicon-heterojunction tandem solar cells processed at low temperature. Energy Environ. Sci. **9**, 81–88 (2016). https://doi.org/10.1039/C5EE02965A

[90] S. Zhu, X. Yao, Q. Ren, C.C. Zheng, S. Li, Y. Tong, B. Shi, S. Guo, L. Fan, H. Ren, C. Wei, B. Li, Y. Ding, Q. Huang, Y. Li, Y. Zhao, X. Zhang, Nano Energy **45**, 280–286 (2018). Transparent electrode for monolithic perovskite/silicon-heterojunction two-terminal tandem solar cells. https://doi.org/10.1016/j.nanoen.2017.12.043

[91] J.Y. Hyun, K.M. Yeom, S.-W. Lee, S. Bae, D. Choi, H. Song, D. Kang, J.-K. Hwang, W. Lee, S. Lee, Y. Kang, H.-S. Lee, J.H. Noh, D. Kim, Perovskite/silicon tandem solar cells with a *V*_OC_ of 1784 mV based on an industrially feasible 25 cm^2^ TOPCon silicon cell. ACS Appl. Energy Mater. **5**, 5449–5456 (2022). https://doi.org/10.1021/acsaem.1c02796

[92] J. Werner, A. Walter, E. Rucavado, S.-J. Moon, D. Sacchetto, M. Rienaecker, R. Peibst, R. Brendel, X. Niquille, S. De Wolf, P. Löper, M. Morales-Masis, S. Nicolay, B. Niesen, C. Ballif, Zinc tin oxide as high-temperature stable recombination layer for mesoscopic perovskite/silicon monolithic tandem solar cells. Appl. Phys. Lett. **109**, 233902 (2016). https://doi.org/10.1063/1.4971361

[93] Z Song, J. Werner, N. Shrestha, F. Sahli, S. De Wolf, B. Niesen, S.C. Watthage, A.B. Phillips, C. Ballif, R.J. Ellingson, M. J. Heben, Probing photocurrent nonuniformities in the subcells of monolithic perovskite/silicon tandem solar cells. J. Phys. Chem. Lett. **7**, 5114–5120 (2016). https://doi.org/10.1021/acs.jpclett.6b02415

[94] J.P. Mailoa, C.D. Bailie, E.C. Johlin, E.T. Hoke, A.J. Akey, W.H. Nguyen, M.D. McGehee, T. Buonassisi, A 2-terminal perovskite/silicon multijunction solar cell enabled by a silicon tunnel junction. Appl. Phys. Lett. **106**, 121105 (2015). https://doi.org/10.1063/1.4914179

[95] K.H. Kim, C.S. Park, J.D. Lee, J.Y. Lim, J.M. Yeon, I.H. Kim, E.J. Lee, Y.H. Cho, Record high efficiency of screen-printed silicon aluminum back surface field solar cell: 20.29%. Jpn. J. Appl. Phys. **56**, 08MB25 (2017). https://doi.org/10.7567/JJAP.56.08MB25

[96] V. Meemongkolkiat, K. Nakayashiki, D.S. Kim, R. Kopecek, A. Rohatgi, Factors limiting the formation of uniform and thick aluminum–back-surface field and its potential. J. Electrochem. Soc. **153**, G53−G58 (2006). https://doi.org/10.1149/1.2129106

[97] S. Narasimha, A. Rohatgi, A.W. Weeber, An optimized rapid aluminum back surface field technique for silicon solar cells. IEEE Trans. Electron Devices, **46**, 1363−1370 (1999). http://doi.org/10.1109/16.772477

[98] B.G. Priyadarshini, A.K. Sharma, Design of multi-layer anti-reflection coating for terrestrial solar panel glass. Bull. Mater. Sci. **39**, 683–689 (2016). https://doi.org/10.1007/s12034-016-1195-x

[99] H.K. Raut, V.A. Ganesh, A.S. Nair, S. Ramakrishna, Anti-reflective coatings: A critical, in-depth review. Energy Environ. Sci. **4**, 3779–3804 (2011). https://doi.org/10.1039/C1EE01297E

[100] Q. Lin, A. Armin, R.C.R. Nagiri, P.L. Burn, P. Meredith, Electro-optics of perovskite solar cells. Nat. Photonics **9**, 106–112 (2015). https://doi.org/10.1038/nphoton.2014.284

[101] S. Jung, K.-Y. Kim, Y.-I. Lee, J.-H. Youn, H.-T. Moon, J. Jang, J. Kim, Optical modeling and analysis of organic solar cells with coherent multilayers and incoherent glass substrate using generalized transfer matrix method. Jap. J. Appl. Phys. **50**, 122301 (2011). https://doi.org/10.1143/JJAP.50.122301
